# The Zebrafish Amygdaloid Complex – Functional Ground Plan, Molecular Delineation, and Everted Topology

**DOI:** 10.3389/fnins.2020.00608

**Published:** 2020-07-16

**Authors:** Baylee A. Porter, Thomas Mueller

**Affiliations:** ^1^Division of Biology, Kansas State University, Manhattan, KS, United States; ^2^Department of Biochemistry and Molecular Biology, Department of Urology, SUNY Upstate Medical University, Syracuse, NY, United States

**Keywords:** telencephalon, teleost, amygdala, hippocampus, isocortex, emotion, prefrontal cortex, prethalamic eminence

## Abstract

In mammals and other tetrapods, a multinuclear forebrain structure, called the amygdala, forms the neuroregulatory core essential for emotion, cognition, and social behavior. Currently, higher circuits of affective behavior in anamniote non-tetrapod vertebrates (“fishes”) are poorly understood, preventing a comprehensive understanding of amygdala evolution. Through molecular characterization and evolutionary-developmental considerations, we delineated the complex amygdala ground plan of zebrafish, whose everted telencephalon has made comparisons to the evaginated forebrains of tetrapods challenging. In this radical paradigm, thirteen telencephalic territories constitute the zebrafish amygdaloid complex and each territory is distinguished by conserved molecular properties and structure-functional relationships with other amygdaloid structures. Central to our paradigm, the study identifies the teleostean amygdaloid nucleus of the lateral olfactory tract (nLOT), an olfactory integrative structure that links dopaminergic telencephalic groups to the amygdala alongside redefining the putative zebrafish olfactory pallium (“Dp”). Molecular characteristics such as the distribution of substance P and the calcium-binding proteins parvalbumin (PV) and calretinin (CR) indicate, that the zebrafish extended centromedial (autonomic and reproductive) amygdala is predominantly located in the GABAergic and *isl1*-negative territory. Like in tetrapods, medial amygdaloid (MeA) nuclei are defined by the presence of substance P immunoreactive fibers and calretinin-positive neurons, whereas central amygdaloid (CeA) nuclei lack these characteristics. A detailed comparison of *lhx5*-driven and *vGLut2a*-driven GFP in transgenic reporter lines revealed ancestral topological relationships between the thalamic eminence (EmT), the medial amygdala (MeA), the nLOT, and the integrative olfactory pallium. Thus, the study explains how the zebrafish amygdala and the complexly everted telencephalon topologically relate to the corresponding structures in mammals indicating that an elaborate amygdala ground plan evolved early in vertebrates, in a common ancestor of teleosts and tetrapods.

## Significance

Based on molecular and evolutionary-developmental characteristics, the study identifies the elaborate amygdala ground plan in zebrafish and stresses the evolution of a complex emotional system in early vertebrates. A multinuclear forebrain structure, the amygdala of mammals has been viewed as a requirement for sophisticated emotions, social behavior, and emotional sentience. Comparable emotional phenomena are rarely discussed in fish in accordance with the current consensus that their amygdala is rather rudimentary and incomparable. Despite obvious morphological differences, however, we show that considerable ancestral amygdaloid building blocks are shared between fish and tetrapods including mammals. The study introduces a long-needed testable molecular reference paradigm of the mature zebrafish extended amygdaloid complex studying neural underpinnings and evolution of emotion in this important model organism.

## Introduction

**FIGURE 1:**
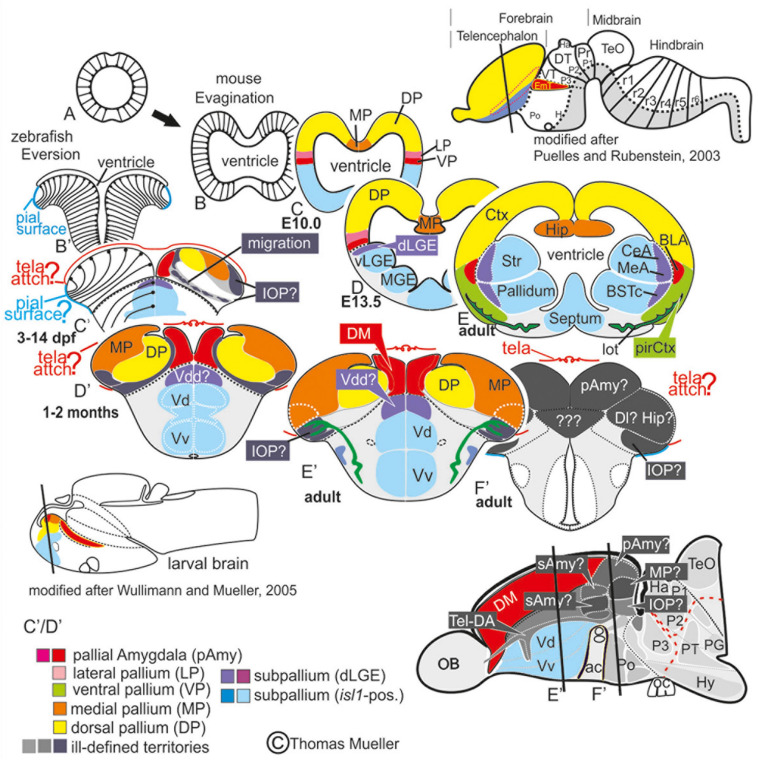
Telencephalic eversion in zebrafish and comparison to mammals. The schematic illustrates how both the outward-growing (eversion) process of the developing telencephalon and its adult morphology of zebrafish (lower row) compares to the telencephalon development (evagination) of mammals (upper row). **(A)** The telencephalon develops from the anteriormost part of the neural tube. **(B–E)** In mammals, two bilateral hemispheres develop around a centrally located ventricle. Predominantly glutamatergic pallial zones (warm colors) develop in the dorsal telencephalon, whereas mostly GABAergic subpallial territories (cold colors) are found in the ventral telencephalon. **(B’–E’)** Likewise, in teleosts like zebrafish, the dorsal and ventral telencephalon, respectively, hold pallial and subpallial territories, however, the ventricle due to the eversion comes to lie on top of the brain. **(C’,D’)** Proliferation patterns, BrdU-long term labeling, and gene expression studies in zebrafish indicated a complex eversion process that includes a radial migration toward the dorsoposterior pallial (“Dp”) zone (Mueller et al., 2011). The zebrafish dorsal pallium (yellow) subsequently gets overgrown by the pallial amygdala and the medial pallium (MP in orange). **(F’)** The teleostean eversion process is not comprehensively understood. A major obstacle in the comprehensively understanding both the eversion and comparative anatomy remains the unsolved delineation of the integrative olfactory pallium (IOP – the putative homolog to the mammalian piriform cortex) and of telencephalic entities in the posteriormost extent. Ambiguous structures and focus of this study are colored in gray.

In mammals and other tetrapods more than a dozen telencephalic nuclei form the amygdala, the regulatory core of the emotional brain (Swanson and Petrovich, 1998; de Olmos and Heimer, 1999; Abellan et al., 2013). The early evolution of a complex amygdala, its organization and behavioral significance in basally derived vertebrates are poorly understood. Until now this heterogeneous structure, the key to emotion and social behavior, remained ill-defined in ray-finned fish (actinopterygii), because their telencephalon looks markedly different from the familiar mammalian situation. In these fish a complex developmental outgrowth called eversion (Figure 1) leads to a topographic rearrangement of forebrain territories relative to non-actinopterygian vertebrates (Wullimann and Mueller, 2004b; Northcutt, 2008; Nieuwenhuys, 2009b; Mueller et al., 2011). To this day, scientists have failed to consistently map pallial and subpallial (subcortical) territories even in relatively well-investigated teleost fish such as cichlids and cyprinids (carp-like fish) like goldfish or zebrafish. As a result, putative homologies across teleosts remain persistently debated (Elliott et al., 2017; Yamamoto et al., 2017) owing to inconsistencies in terminology, lack of robust molecular demarcations of brain nuclei, and erroneous annotations of molecular expression patterns.

We unraveled the zebrafish amygdala using a holistic approach that integrates molecular and chemoarchitectonic characteristics and previously published developmental data with a neuroethological framework that adopts the concepts of “the *extended amygdala”* and *“primary olfactory cortex”* originally developed by examining macrosmatic rodents that is, mammals with a pronounced sense of smell (Swanson and Petrovich, 1998; de Olmos and Heimer, 1999). The extended amygdala concept considers numerous subpallial (subcortical) regions as part of the amygdala, including subdivisions of the bed nucleus of the stria terminalis (BST) and the centromedial (CeA, MeA) nuclei. These additional territories extend the classic definition of the amygdala, which included exclusively pallial (cortical) nuclei such as the basolateral amygdala (BLA) critical for fear conditioning (Swanson and Petrovich, 1998). The concept of the primary olfactory cortex includes all pallial nuclei that receive projections from olfactory bulb neurons, several of which form part of the mammalian amygdala. For example, the posteromedial cortical nucleus (PMCo) and the composite nucleus of the lateral olfactory tract (nLOT) are amygdaloid nuclei essential for olfactory cued behavior. Usually, the entire amygdala is described as a heterogeneous collection of predominantly olfactory pallial and subpallial territories that regulate emotion and autonomic nervous system function (Swanson and Petrovich, 1998). In addition, analyzing the distributions of *lhx5*- and *vGLut2a*-driven green fluorescent protein (GFP) in the transgenic lines *tg(lhx5:GFP)* and *tg(vGlut2a:GFP)* we uncovered the ancestral relationships between the everted olfactory pallium and the thalamic eminence (EmT).

Our analysis, therefore, capitalizes on the deep evolutionary relationship between EmT, amygdala, and the sense of smell. Like rodents, zebrafish are macrosmatic animals whose reproductive behavior is hugely influenced by pheromones (Lazzari et al., 2014). Male zebrafish exposed to the female sex pheromone prostaglandin F2α (PGF2α) express stereotypic nudging behavior and conspecific male-male aggression (Sorensen et al., 1988; Yabuki et al., 2016). Molecular data in amphibians (frogs and salamanders) and sauropsids (birds and reptiles) have established a conserved amygdala blueprint across tetrapods (Martínez-García et al., 2007; Medina et al., 2011, 2017). For teleosts like zebrafish the amygdala remained vaguely defined (Wullimann and Mueller, 2004b; Northcutt, 2008; von Trotha et al., 2014; Ruhl et al., 2015). This lack of a comprehensive amygdala systems understanding hindered the study of the neural mechanisms underlying cognition, emotion, and social behavior in teleosts and hampered comparisons to mammals and other tetrapods. Our study, thus, introduces a long-needed and testable reference paradigm of the zebrafish amygdala’s functional organization, molecular definition, and evolution.

## Materials and Methods

### Fish Maintenance and Stocks

We keep zebrafish (*Danio rerio*, Cyprinidae) in a LACS operated facility at Kansas State University (KSU) in Manhattan under standard conditions at 28°C (Westerfield, 2000). The University Institutional Animal Care and Use Committee (IACUC) reviewed and approved all experimental protocols of this study. Our experiments conform to the NIH Guidelines for the Care and Use of Laboratory Animals. We used four previously published transgenic lines: (1) *Tg(isl1:GFP)* (Higashijima et al., 2000); (2) *Tg(vGlut2a:GFP)* also named *Tg(slc17a6b:EGFP)* (Bae et al., 2009); (3) *Tg(lhx2a:GAP-YFP)* (Miyasaka et al., 2009), and *Tg(lhx5:GFP)* (Peng and Westerfield, 2006; Turner et al., 2016).

### Immunohistology

Immunohistology on cryosectioned brain sections was performed as described (Rink and Wullimann, 2001). We used only antibodies with previously validated specificity including rabbit anti-calretinin (Swant, catalog# 7697/1:1000), mouse anti-parvalbumin (Millipore 1:5000), rabbit anti-γ-aminobutyric acid (GABA, Sigma, catalog# A2052, 1:5000), rabbit anti-substance P (SP; immunostar, catalog# 20064/lot#1003002, 1:2000), mouse anti-tyrosine hydroxylase (TH; 1:1000; catalog# 22941; lot# 1241002) Immunostar; catalog# 20066; lot# 1301001), 1:1000), chicken anti-GFP (1:1000; molecular probes/invitrogen, catalog# A10262; lot# 1729643), rabbit anti-GFAP (1:100, Immunostar), rabbit anti-DSRed antibody (Living Colors, Clontech, Cat# 632496, 1:1000). Secondary fluorescence-coupled antibodies (Invitrogen): goat anti-chicken Alexa Fluor 488, goat anti-rabbit Alexa Fluor 488/555, goat anti-mouse Alexa Fluor 488/555. We used a total of 65 adult (unsexed) zebrafish fish and stained with maximally three antibodies at the same time so that each pattern was represented by at least three samples.

### Confocal and Conventional Epi-Fluorescence Microscopy and Image Analysis

To image the distribution patterns of fluorescence immunohistologically stained neuronal phenotype patterns we routinely used a ZEISS Axioplan-2 fluorescence microscope, a confocal Zeiss 700 microscope (microscope core facility KSU), and an Olympus automated epi-fluorescence microscope. Post imaging techniques included montaging, stitching, and sharpening techniques using Slidebook 6 and Adobe Photoshop. We used CorelDraw X7/8 for labeling anatomical structures and the generation of photoplates and figures.

### Approach and Redefined Terminology

First, we generated a molecular atlas of the zebrafish telencephalon by imaging and analyzing numerous immunohistologically stained sections of adult zebrafish brains (both wildtype and transgenic lines. Specifically, we compared GFP distribution in reporter lines [*Tg(vGlut2a:GFP*, *Tg(isl1:GFP, Tg(lhx2a:GAP-YFP*), *Tg(lhx5:GFP]* in fluorescence immunostained cross sections of the telencephalon of adult fish that were additionally stained against GABA- and tyrosine hydroxylase (TH, a marker for dopaminergic neurons in the zebrafish forebrain), the neuropeptide substance P, and the calcium binding proteins parvalbumin and calretinin. We stained against up to three antigens simultaneously and always used DAPI as counterstain. This systematic multi-marker approach identifies amygdaloid nuclei based on the distribution of neurochemically defined neurons, gene expression patterns, and fiber courses that reflect topological relationships. In addition, we integrated published data on the organization and development of the zebrafish telencephalon (Castro et al., 2006; Mueller et al., 2008, 2011; Mueller and Guo, 2009; Mueller and Wullimann, 2009; Ganz et al., 2012; Ruhl et al., 2015; Lal et al., 2018). Moreover, we build on an earlier study regarding the putative isocortex-homolog (dorsal pallium) that stressed a complex pallial eversion (Figure 1) because our new data strongly support this previously proposed model (Mueller et al., 2011).

Similar to former studies and in accordance with established molecular data in zebrafish and functional findings in goldfish, we use the term medial pallium for the dorsolateral pallial zone of zebrafish (Dl) (Ganz et al., 2012; Ocana et al., 2017; Rodriguez-Exposito et al., 2017). To provide a meaningful framework and further improve conceptual understanding, we adapted vocabulary for the zebrafish amygdala to the terminologies used for amphibians and mammals. This is not to say that our comparison between similar molecular compositions in zebrafish and mammals suggests simple one to one equations. While we focus on comparing zebrafish to mammals, amygdaloid nuclei in this latter group are more diversified and different subdivisions can show similar molecular compositions. Mammalian amygdaloid territories, of course, also show species-specific differences and a greater cell type variety than zebrafish at large. Our modified terminology treats newly demarcated amygdaloid territories as potentially field homologs to one or more nuclei within tetrapod species and we indicate specific proposed homologies where necessary within Table 1. Instead of individual nuclei, we focused on the identification of common building blocks critical for understanding the underlying organizational scheme. Supplementary Table S1 lists former topographical terminologies for goldfish and zebrafish and their labeling inconsistencies.

**TABLE 1:** The zebrafish *extended amygdala* and *primary olfactory pallium* including putative homologies to mammals.

Probable major developmental origin	dLGE					Septum (subpallial)		vLGE (striatal)	EmTr	MGE/pallidal	pAmy	pAmy	EmT-d +DP(?)	LP

Anatomical/Structures Marker	CeAa	CeAl	CeAd *(+IC)*	BSTa	BSTp	MeAa	MeAd	MeAv *(MeApd)*	MeAp (*MeApv*)	BSTm	DM	PMPa *(PMCo)*	nLOT	IOP
Topographical location	Vdd	Vc	Vdd	T-DA	T-DA	Vdd	Vp	Vs	Vi	Vs	Dm	Dm	Dlv/Dp	Dl(p)
*Tg(isl-1*:GFP)	−−	−−	−−	−−	−−	−−	−−	Sparse	−−	++	−−	−−	−−	−−
Tyrosine hydroxylase (TH)	−−	−−	−−	++	++	−−	−−	+	−−	−−	−−	−−	Fibers	−−
GABA	++	++	++	++	++	++	++	++	+	++	Sparse	Sparse	1 layer	Sparse
Parvalbumin	+	–	++	+	+	−	+	+	+	−−	−−	−−	+	++
Calretinin	−−	−−	−−	+	+	+	++	++	+	+	+	++	−−	−−
substance P (SP)	−−	−−	−−	−−	−−	+	+	+	Sparse	−−	Fibers	+	−−	+
Otpa	−−	−−	−−	−−	−−	−−	−−	–	++	−−	−−	−−	−−	−−
*Tg(lhx2a:GAP-YFP)*	−−	–	−−	−−	−−	−−	−−	Fibers	Fibers	Fibers	−−	−−	Fibers	Fibers
*Tg(gad1b:dsRed)*	+	–	−−	+	+	+	−	+	Sparse	+	−/+	−/+	−/+	−/+
*Tg(vGlut2a:GFP)*	−−	–	−−	−−	−−	−−	−−	−−	++	−−	++	+	−−	+
*Tg(lhx5:GFP)*	−−	−−	−−	−−	−−	−−	−−	−−	++	−−	−−	−−	−−	Fibers

	**Extended amygdala/subpallial nuclei plus EmT(r)**										**Pallial nuclei**			

*Molecular definitions, characteristic neuron types, and topological fiber courses differentially labeling candidate extended amygdala, nucleus of the lateral olfactory tract, and homolog to the mammalian lateral pallium (LP, entorhinal cortex, claustrum and insula) in zebrafish. Nomenclature is adapted to amphibian and mammalian terminologies to facilitate a functional systems understanding. Classical terminologies for the topographical location in the teleostean telencephalon are listed above. Note that oftentimes the part of the region “Vdd” that we consider as a GABAergic subpallial territory is mislabeled as “Dm-v” by some authors. Putative mammalian homologs in italics. For abbreviations see list of abbreviations.*

Of course, we build on previous knowledge that established basic functional and hodological similarities to tetrapod amygdaloid nuclei, (1) the teleostean dorsomedial pallial zone (“Dm”) most often viewed as the pallial amygdala and (2) three subpallial candidate amygdaloid nuclei typically termed supra- and postcommissural nuclei (Vs, Vp, BST) (Braford, 1995; Portavella et al., 2004a; Wullimann and Mueller, 2004b; Northcutt, 2008; Ganz et al., 2014). Here, we provide additional data that either confirm, establish, redefine, or extend classical definitions of these nuclei; we discuss these in the text when needed. Last but not least, we apply the widely accepted prosomeric model as a paradigm for comparing the forebrains zebrafish and mammals (Wullimann and Puelles, 1999; Puelles and Rubenstein, 2003, 2015; Wullimann and Mueller, 2004a). Developmental studies indicated that the topological anterior tip of the prosomeric forebrain is the optic chiasm and the alar-plate derived telencephalon consists of a topologically anterior subpallium and a posterior pallium (Puelles and Rubenstein, 2003). In classical columnar paradigms of the forebrain, these entities have been mis-represented as ventral (subpallial) and dorsal (pallial) portions of the telencephalon, with the olfactory bulbs as the (topographical) anterior tip of the brain (Puelles, 2019; Puelles et al., 2019). To accommodate our terminologies to these two different perspectives, we use the terms “anterior” and “posterior” for topological annotations from the perspective of the prosomeric model. In contrast, we use “rostral” and “caudal” to refer to topographical locations in orientation to either the nose or the tail. However, as an exception to this rule, we used the terms anterior, ventral, dorsal, and posterior in the classical (topographical) sense for most amygdaloid territories simply to better relate them to similarly named structures in tetrapods.

Our iterative strategy solved the complexities of the zebrafish amygdala by starting with the identification of the nLOT and most readily identifiable nuclei, such as the medial extended amygdaloid nuclei (MeAa, MeAv, MeAd, and MeAp), and moving to more obscure regions, such as the posteromedial pallial nucleus (PMPa) most likely homologous to the mammalian posteromedial cortical nucleus (PMCo), and the anterior, lateral and dorsoposterior central amygdaloid nuclei (CeAa, CeAl, and CeAd). In addition, we propose that the dopaminergic subpallial groups belong to the newly defined anterior and posterior divisions of the bed nucleus of the stria terminalis (BSTa, BSTp). Within the new paradigm, the nLOT and newly identified rostral, medial, and lateral portions of the thalamic eminence (EmTr, EmTm, and EmTl) form pivotal landmarks for topologically relating the everted zebrafish amygdala to the evaginated mammalian telencephalon. We identified the heterogenous nLOT based on its proximity and partial convergence with thalamic (EmT and Emtl) territories within the region of the diencephalic-telencephalic junction (DTJ) as defined in this study. In fact, we consider the identification of the EmT derivatives together with the characterization of the DTJ and as pivotal for understanding the everted topology of the teleostean telencephalon. Previously, the nLOT has been misunderstood as the dorsoposterior olfactory pallium (Dp) and highest integrative olfactory center in zebrafish. We, instead, show that the highest olfactory pallial territory occupies a region that is currently considered the posteriormost part of the dorsolateral zone (Dl) most often assigned to the “hippocampal” medial pallium. As a result, we named this posterior dorsolateral zone the “integrative olfactory pallium (IOP),” which we assume is homologous to the lateral pallium (LP) and functionally comparable to the mammalian entorhinal cortex that forms part of the hippocampal formation (Watson and Puelles, 2017). The precise anatomical demarcation and molecular definitions of both the IOP and the nLOT refutes current theories on the functional organization of the olfactory pallial zones (Jacobson et al., 2018). Likewise, the GFAP and *lhx5*-driven GFP data presented do not support simple telencephalic eversion models (Butler, 2000; Nieuwenhuys, 2009a, b; Furlan et al., 2017; Yamamoto et al., 2017). Also, they are not compatible with an incomplete or partial pallial eversion as suggested earlier (Wullimann and Mueller, 2004b). Instead they indicate that the telencephalic outward-growing process is complete and complex and probably constrained by hem-like organizing centers and the thalamic eminence and its derivatives.

## Results

### Overview

Based on the distribution of molecularly defined neuronal phenotypes and calcium binding proteins (calretinin, parvalbumin) that reveal both territory-specific cell type abundances and intra-telencephalic connections, we identify thirteen amygdaloid territories (Table 1). Following established conventions in the field, Table 1 divides predominantly GABAergic subpallial from predominantly glutamatergic pallial structures (Puelles et al., 2000; Mueller et al., 2006) as well as the predominantly glutamatergic MeAp which we consider a thalamic eminence derivative (EmT-d). Due to its dual nature as being both a part of the medial extended amygdala and derivative of the rostral otp-a positive thalamic eminence (EmTr), we refer to this structure as “MeAp/EmTr.”

Table 1 highlights four key findings:

(1) Most of the subpallial amygdaloid nuclei in zebrafish (CeAa, CeAl, BSTa, BSTp, and MeAd) form part of the GABAergic, *isl1:*GFP-free region that we propose corresponds to or develop within the region of the dorsal lateral ganglionic eminence (dLGE); not to the “dorsal striatum” as previously suggested (Ganz et al., 2012). The *isl1:GFP* positive BSTm represents an extension of the striatopallidal systems, that is the teleostean counterpart of the tetrapod medial ganglionic eminence (MGE) that gives rise to the pallidum consistent with a former study that identified this nucleus (Ganz et al., 2012) as well as a study that confirmed its pallidal identity (Wullimann and Umeasalugo, 2020). In contrast, we interpret the newly identified MeAv, despite its absence of *isl1*-driven GFP, as an extension of the striatum proper based on the presence of both substance P and calretinin cells (this study). Notably, we consider the predominantly glutamatergic MeAp a derivative of the rostral EmT (MeAp/EmTr). This structure has been previously viewed as the “MeA” in zebrafish and was termed intermediate nucleus of the subpallium (“Vi”) (Biechl et al., 2017). In contrast, we show that this nucleus is predominantly glutamatergic and contiguous or identical with the thalamic eminence located within what we consider the diencephalic telencephalic junction (DTJ).
(2) The presence of numerous calretinin positive neurons define medial amygdaloid nuclei (MeAd, MeAv, MeAp) whereas the lack of pronounced populations of calretinin expressing neurons is indicative of central amygdaloid nuclei (CeAa, CeAl, and CeAd) similar to the mammalian situation at early stages of development (Wojcik et al., 2013). The CeAd stands out as a laterally displaced subpallial (GABAergic) nucleus that is defined by the presence of dense parvalbumin expressing fibers and some parvalbumin-expressing neurons.
(3) The study identifies a territory in the caudalmost position of the former dorsomedial zone (“Dm”) as the putative homolog of the mammalian posteromedial cortical amygdaloid nucleus (PMCo), based on substance P-fibers passing both through both the medial amygdala and posterior Dm. We termed this territory the “posteromedial pallial amygdaloid zone” (PMPa), its homology to the mammalian PMCo is also supported by its intercalated position between MeAd and integrative olfactory pallium (IOP) and many calretinin positive neurons, which potentially share developmental origins with those populating medial amygdaloid territories.
(4) We used the transgenic line *Tg(lhx2a:GAP-YFP*) to distinguish the newly identified nucleus of the lateral olfactory tract (nLOT) from the principal olfactory pallium (Dp proper), which we termed in zebrafish the “integrative olfactory pallium” (IOP) owing to its prospective higher integrative function. The heterogenous amygdaloid nucleus of the former olfactory tract (nLOT) was previously mislabeled as the dorsoposterior pallial zone (“Dp”) and most likely serves olfactory and taste integration at a primary (lower) level. We propose that this composite structure in zebrafish is homologous with the mammalian nLOT, because—like its mammalian counterpart— it consists of pallial (glutamatergic), subpallial (GABAergic) and (glutamatergic) putative thalamic derivatives (EmT-d) (Huilgol and Tole, 2016). Most importantly, we show that the lateral thalamic eminence (EmTl) form the base of the nLOT and *lhx5*-driven GFP positive cells are present in the posterior lateral olfactory tract territory (pLOT) strongly resembling the mammalian situation during development (Ruiz-Reig et al., 2017). Again, as in mammals, a stream of migrating pallial neuroblasts originating from a region close to or overlapping with the pallial amygdala form part of this region in zebrafish (Remedios et al., 2007; Mueller et al., 2011). In addition, *lhx5*-driven positive elements are found within and close to Dp (Turner et al., 2016). Our results suggest that these elements represent derivatives of the thalamic eminence that, like their counterparts in mammals, may both control and contribute to the development of the nLOT and other structures at the DTJ in zebrafish. Interestingly, the heterogenous nLOT in zebrafish receives both gustatory and main olfactory input (Miyasaka et al., 2009; Yanez et al., 2017) further supporting its functional and structural heterogeneity.

## Pallial and Subpallial Derivatives in the Anterior Telencephalon

### Pallial Amygdala (pAmy)

A recent study on the developing zebrafish pallium suggests a concentric pallial growth and simple eversion (Furlan et al., 2017) as similarly postulated before (Butler, 2000; Nieuwenhuys, 2009b). This hypothesis strongly conflicts with earlier findings that showed a complex pallial eversion and identified the dorsal pallium (DP) based on molecular distinctiveness and topological relationships to two of the major pallial territories, the putative pallial amygdala [i.e., the dorsomedial (Dm) zone] and the medial pallium (MP; Figure 1; Mueller et al., 2011). A solid demarcation of these pallial histogenetic units is pivotal for the complete understanding of the pallial and subpallial territories forming the entire amygdaloid complex, which is the objective of this study.

To solve conflicting views, we tested if additional molecular characteristic set the prospective dorsal pallium (DP) apart from the medial pallium (MP; roughly the dorsolateral (Dl) pallial zone in zebrafish) and the putative pallial amygdala (the dorsomedial (Dm) zone). We determined that the distribution of specific glutamatergic neurons visualized by GFP in the transgenic line *Tg(vGlut2a:GFP)* in comparisons to parvalbumin and GABA strongly supported the molecular distinctiveness and delineation of the dorsal pallium candidate. Focusing on these chemoarchitectonic characteristics, we were able to corroborate that the centralized portion of the prospective dorsal pallium is covered by the molecularly distinct DM and medial pallium (MP) at posterior sections as previously suggested (Mueller et al., 2011).

**FIGURE 2:**
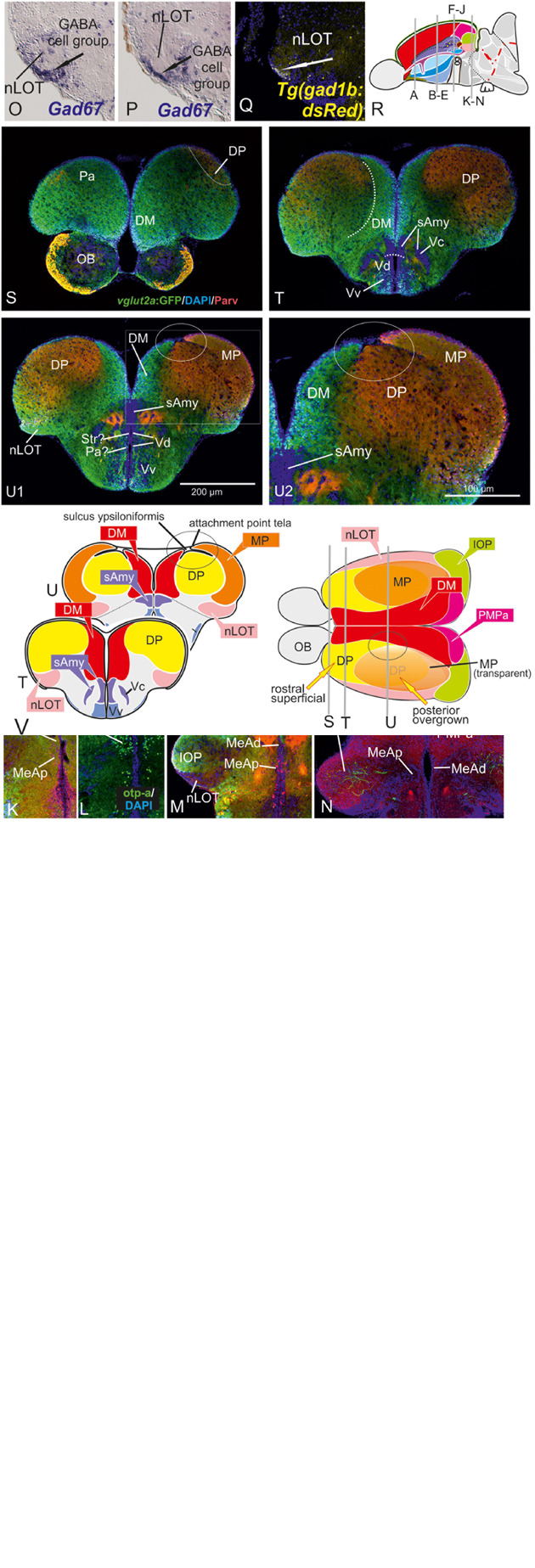
Molecular Definitions of the Zebrafish Amygdaloid Complex. Precommissural **(A–E)**, supra- and postcommissural **(F–Q)** telencephalon, and delineation of the dorsal pallium in its periventricular {(S+T, U1/left) and central (U1, right, U2)} portions. Pallium: Analyzing the distributions of GABA **(A–C,H,K)**, parvalbumin **(E,F,J,N)**, and GFP in the transgenic line *Tg(vGlut2a:GFP)* **(A–C,H,I,K,M)** allowed to discern the dorsomedial pallial zone, the putative core region of the pallial amygdala, from the hippocampal division (medial pallium/MP) and the teleostean dorsal pallium (DP) **(A–C)**. DP lacks both *vGlut2a*-driven GFP and parvalbumin-positive neurons but shows parvalbumin positive fibers **(F,J,H,U2)**. The strongly GFP-positive DM lacks parvalbumin expression **(F,J)**. The mostly *vGlut2a*:GFP-free hippocampal division (MP) exhibits both parvalbumin-positive fibers and neurons in addition to GABA-neurons **(C,F,J)**. Only at posteriormost sections, we found *vGlut2a*:GFP positive neurons in the MP **(H)**. At anteriormost sections **(A,B)**, the DP reaches the dorsalmost periventricular zone, which holds proliferative stem cells. More posteriorly **(C,F,J)**, the DP is shown in its secondarily centralized position overgrown by both the pAmy and hippocampal division (medial pallium/MP). In addition, we found many large GABAergic interneurons in DP **(A–C)**, as well as sparsely distributed GABAergic interneurons in the pAmy and MP **(B,C)**. Subpallium: Dense population of GABAergic neurons define the subpallium **(A–C,H,K)**. Within the subpallium, *isl1*-driven GFP labels striatopallidal and septal divisions as well as the BSTm **(D1,D2,F,G)**. In contrast, GABAergic territories that lack *isl1*-driven GFP define the majority of subpallial amygdaloid territories. The anterior (precommissural) dorsalmost. GABAergic population (CeAa) together with the migrated GABAergic nuclei (CeAl = former Vc) predominantly form the zebrafish central amygdala [CeA in **(A–C)**]. The ventricular-close GABAergic territory is juxtaposed and contiguous with the former supracommissural (Vs) and postcommissural (Vp) nuclei of medial amygdala (MeA; former Vs/Vp). In addition, based on the lack of *isl1*-driven GFP in both anterior and posterior TH-positive dopaminergic and their previous reported projection to the hypothalamus, we consider as the anterior and posterior bed nucleus of the stria termalis (BSTa/BSTpd). The medial bed nucleus of the stria terminalis (BSTm) is the only *isl1*:GFP positive medial nucleus [BSTm in **(F,G)**]. We also found sparse dopaminergic neurons within the posteromedial amygdala (MeAp) that project into part of the nLOT **(B2)**. Supra- and postcommissural extended medial amygdaloid nuclei (MeAc, MeAd, MeAp, BSTm, BSTpd) are defined by the presence of numerous calretinin-positive neurons **(F,I,M)** similar to the mammalian situation. In contrast, the CeAd that comprises numerous laterally displaced GABAergic neurons shows many parvalbumin-positive fibers and few parvalbumin neurons, but lacks calretinin neurons. The MeAp was formerly assigned to the teleostean subpallium, because there are GABAergic neurons in its vicinity **(K)**. However, the otp-a **(L)** and calretinin **(I,M)** positive neurons that define the MeAp are likely glutamatergic and originate from the thalamic eminence according to our results. Discerning PMPa, IOP, and nLOT Comparing secondary olfactory projections in the transgenic line *Tg(lhx2a:GAP-YFP)* **(J,N)** with parvalbumin allowed us to identify primary olfactory pallial territories and their topological relationships. The distribution of secondary olfactory projections in comparison to parvalbumin and calretinin fibers identifies two olfactory integrative structures: (1) the parvalbumin-positive integrative olfactory pallium (IOP) **(I,J,M,N)** formerly misinterpreted as part of the medial pallium (“Dl/Dlp”) in zebrafish; (2) the largely parvalbumin-free (migrated) amygdaloid nucleus of the lateral olfactory tract (nLOT). The nLOT is a predominately glutamatergic nucleus that consists of both *vGlut2a*:GFP positive (anterior; **C**) and negative (posterior) domains as well as *GAD67*-positive [arrows in **(O,P)**] and *gad1b*-driven dsRed expressing [arrow in **(Q)**] GABAergic zones resembling the mammalian nLOT that consists of alternating GABAergic and glutamatergic layers. Previously, the nLOT has been mislabeled as the posterior division of the dorsal telencephalon (“Dp”) and mistakenly considered the piriform cortex homolog. The identification of the nLOT together with the piriform cortex homolog (IOP) solves their topological relationships within the complexly everted telencephalon (R indicates orientation of sections **A–N**). **(F)** Schematic figure shows orientation of section in the sagittal view. New hallmarks of the complexly everted zebrafish telencephalon – the rostral (superficial) versus posterior centralized parts of the dorsal pallium (DP). **(S–U2)** Analyzing distribution of parvalbumin versus *vGlut2a*-driven GFP shows that the dorsal pallium reaches the periventricular zone and includes its germinative layer of origin. Note, that DM and medial pallium (MP = dorsolateral (Dl) zone of the pallium) overgrow the dorsal pallium at posterior sections at the point of convergence (circled area). **(V)** Schematics of a zebrafish telencephalon from dorsal perspective illustrating levels of cross sections.

In the transgenic line *Tg(vGlut2a:GFP)*, we found that GFP is expressed only in subsets of glutamatergic neurons that differentially populated pallial derivatives. In fact, we found *vGlut2a*-driven GFP (*vGlut2a*:GFP) positive neurons heavily labeled the dorsomedial (DM) zone here considered a derivative of the pallial amygdala (pAmy), whereas the putative dorsal pallium (mammalian isocortex) entirely lacked GFP (Figures 2A–C,H,K). This lack of GFP in the dorsal pallium (DP) indicates that DM does not contribute neurons to the development of DP and that they indeed represent distinct histogenetic units. In addition, the hippocampal pallium (MP, medial pallium) showed *vGlut2a*:GFP exclusively in its anterior- and posteriormost extents and dense populations of parvalbumin-positive neurons throughout its entire expansion (Figures 2E,F). The medial pallium thus also represents a distinctive pallial unit that is easily distinguishable from both the parvalbumin-free DM and the dorsal pallium (DP) that showed parvalbumin fibers yet lacked parvalbumin-positive neurons. The further molecular characterization of these distinct pallial units thus validates previous publications on topology (homology), function, and transcription factor profiles of the three major pallial derivatives: (1) the pallial amygdala (DM, PMPa), the dorsal pallium, and (3) the medial pallium (Mueller et al., 2011; Ganz et al., 2012). This expression pattern is also visible in 2 and 3-week-old larval zebrafish (personal observation).

The validation of all of these three pallial entities as distinct developmental entities enabled us to approach the remaining pallial and subpallial entities in the everted telencephalon of zebrafish, especially those that have been controversially discussed (gray zones in Figure 1). Comparing *vGluta2a*:GFP and GABA patterns, we established the ventralmost cells of the *vGlut2a*-GFP positive pAmy as a landmark that defines the (topographically) dorsal side of the pallial-subpallial border (PSB) adjacent to the dense GABAergic and *isl1*:GFP-free cell populations constituting the zebrafish dLGE (Figures 2D1–G). These results indicate that the Dm-region widely viewed as the pallial amygdala (pAmy) in teleosts (Wullimann and Mueller, 2004b; von Trotha et al., 2014; Ruhl et al., 2015), molecularly needs to be subdivided into an anterior part (DM) of the former dorsomedial zone of the pallium (“Dm”) and a posterior part. The presence of predominantly *vGLUT2a*-driven GFP expressing neurons and absence of parvalbumin-fibers and neurons define DM, which we therefore consider a derivative of the pallial amygdala. Notably, a recent study identified a group of so-called Dm120a-neurons as part of DM mediating associative fear learning (Lal et al., 2018). These Dm120a-neurons and most of DM are defined by emx3 expression whereas a posteriomost portion of Dm does not express this gene (Lal et al., 2018). This emx3-negative posteriormost part of the former dorsomedial (Dm) territory according to our results contains the posteromedial pallial nucleus (PMPa) most likely homologous to the mammalian PMCo (this study; Figures 2K–N). In the anterior telencephalon, *vGlut2a*:GFP positive neurons also heavily populate a portion of the nLOT consistent with the hypothesis that this portion of the nLOT is a migrated territory derived from the thalamic eminence located within the DTJ (this study).

### Anterior Extended Amygdala – The Central (CeAa,CeAl) and Anterior Medial Amygdala (MeAa)

In general, dense populations of GABAergic neurons mark the zebrafish striatopallidum (Vd in Figures 2B,C), septal (Vv in Figures 2B,C), anterior (CeAa; Figures 2B,C) and laterally displaced (CeAl) “central amygdaloid” (“Vc”) nuclei (Figures 2B,C). In contrast (and consistent with earlier reports), we detected sparsely distributed GABAergic neurons in pallial territories including the *vGlut2a*:GFP-positive pallial amygdala (pAmy), the olfactory pallial zone (OP), the nucleus of the lateral olfactory tract (nLOT; Figure 2C), as well as the territories that topologically correspond to the mammalian isocortex [=zebrafish dorsal pallium (DP)] and hippocampus [medial pallium (MP)](e.g., Figure 2C).

**FIGURE 3:**
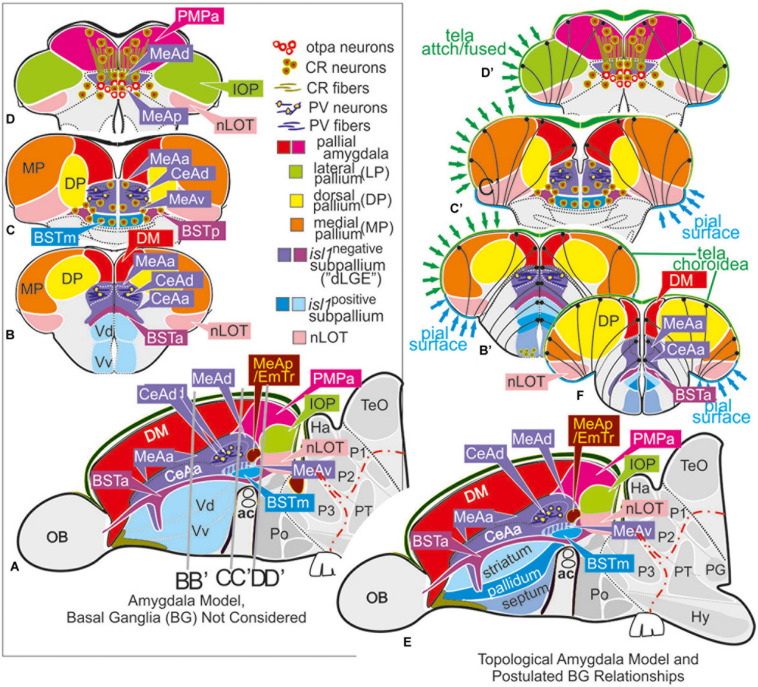
Molecular code of the zebrafish amygdaloid complex. Definitions of amygdaloid territories in the complexly everted telencephalon: Amygdala model (basal ganglia (BG) not considered) **(A–D)** versus idealized topological amygdala model **(B’,C’,D’,E,F)** indicating tela attachments and radial glia distribution. **(A–D)** The lateral schematic **(A)** of the amygdala model divides the zebrafish amygdaloid complex with regard to the anterior commissure (ac) into precommissural **(B)**, supracommissural **(C)**, and postcommissural **(D)** sectors. A hierarchical code defines all amygdaloid nuclei: All medial extended amygdaloid nuclei in the supra- and postcommissural sectors **(C,D)** are located within the *isl1*:GFP negative, subpallial (GABAergic) territories and comprise numerous calretinin-positive neurons. The CeAd is distinguished from such MeA-territories through the presence of parvalbumin-fibers and cells that are laterally displaced. More anterior lying CeA-territories such as the CeAa, CeAl also form part of the *isl1*:GFP negative subpallium, they are, however, distinguished from the CeAd through their lack of parvalbumin expression. In addition, all dopaminergic clusters formerly viewed to the zebrafish striatopallidum are here considered the anterior (BSTa) and posterior (BSTp) divisions of the bed nucleus of the stria terminalis (BST). These dopaminergic extended amygdaloid nuclei are also located in the zebrafish *isl1*:GFP free territory that corresponds to the mammalian dLGE (= Vdd). The medial BST(m) is the only subpallial amygdaloid territory where a large population of *isl1*:GFP forms the majority of this nucleus in addition to some calretinin-positive neurons that link this nucleus with the rest of the extended medial amygdala. The newly discovered integrative olfactory nucleus (IOP) shows secondary olfactory projections and many parvalbumin positive neurons. In contrast, the amygdaloid nLOT that also receives secondary olfactory projections lacks these parvalbumin neurons. **(E)** Topological amygdala model indicating idealized BG relationships showing tela attachment sites and radial glia distribution as revealed in this study.

**FIGURE 4:**
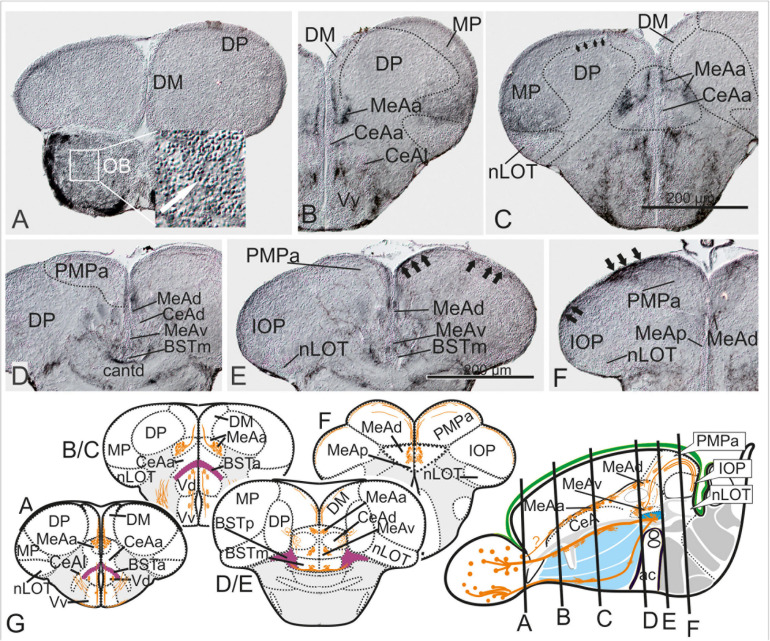
Substance P Fiber Tracts from the Olfactory Bulb into the Telencephalon. **(A–G)** Using diaminobenzidine (DAB) as a substrate for the detection of a horseradish peroxidase coupled antibody yielded enhanced detection of substance P in the telencephalon **(A–G)**. We found substance P positive neurons in the olfactory bulb (OB in A and inlet). substance P fibers emanate from the olfactory bulb toward the lateral ventralmost part of the subpallium [Vv+Vd in **(B,C)**]. Most importantly, some SP cells and many SP fibers label the medial amygdala (MeAa) in the dorsal tier of the subpallium **(B–F)**. At the transition between supracommissural and postcommissural telencephalon **(D–F)**, substance P positive fibers passing through the dorsal medial amygdala [MeAd in **(D–F)**] enter and label the posteromedial pallial amygdala [PMPa in **(E,F)**]. Substance P fibers also pass through the PMCo into the IOP, the zebrafish putative piriform cortex homology (black arrows in IOP of H), supporting the proposed homology between the zebrafish PMPa and mammalian PMCo. Note, the anterior and dorsoposterior divisions of the CeA (CeAa + CeAd) lack substance P and therefore can easily distinguished from all medial amygdaloid nuclei (MeAa, MeAd, MeAv, MeAp). **(G)** Schematic cross sections and sagittal view of the zebrafish telencephalon to illustrate the two solid zebrafish accessory olfactory substance P fiber systems innervating the medial amygdaloid territories. Substance P fibers seemingly form also laterally displaced diffuse projections **(E)**.

The distributions of neuronal phenotype, such as parvalbumin- and TH-expressing dopaminergic neurons was also compared to that of neurons labeled by the transgenic line *Tg(isl1:GFP)* (Figures 2D1–G,J,N). In this way, the *isl1*:GFP negative and GABA-positive subpallial (extended) amygdala was distinguished from positive striatopallidal territories (“Vd”) (Figures 2A–G). Specifically, *isl1*:GFP expressing neurons are confined to a territory ventral to the *isl1*:GFP-negative TH-expressing neurons (Figure 2F). This indicates that the predominantly GABAergic nuclei of the subpallial (extended) amygdala, specifically the CeAa, CeAl, MeAa, and MeAd form the GABA positive and *isl1*:GFP-negative domain sandwiched between the *isl1*:GFP positive striatopallidum and the *vGlut2a*:GFP expressing DM. In other words, the absence of *isl1*:GFP labeled cells marks most of the zebrafish central amygdala (CeAa. CeAl) dorsal to the dopaminergic cell clusters (Cave and Baker, 2009). Only a small territory dorsal to the CeAa belongs to the MeAa, because substance P fibers label this territory as distinct (Figures 4D,E). Thus, the central amygdala (CeA) is defined based on its *isl1*:GFP negativity, its juxtaposed position to both the pAmy and MeAa, and the absence of typical MeA markers such otpa- and calretinin-positive neurons and substance P expression (Figures 2M,L). Figure 3 illustrates how otpa- (Figure 2L) and calretinin-positive neurons (Figure 2M) in the supra- and postcommissural subpallial territories of the BSTm and teleostean dLGE defines the medial extended amygdaloid nuclei as well as the pallial PMPa.

### Dopaminergic Groups Form the Anterior and Posterior Division of the BST

Previous studies have classified telencephalic dopaminergic neurons in teleosts as an integral part of the striatopallidum (Rink and Wullimann, 2001; Tay et al., 2011). However, we found that these dopaminergic neurons are located within *isl1*:GFP-negative territory, in contrast to the *isl1*-driven GFP positive rest of the territory typically labeled as dorsal portion of the subpallium (“Vd”) and considered here the zebrafish striatopallidum. Based on their relative position between *isl1*-negative CeAa, CeAl, and MeA amygdaloid territories and in close proximity to the newly defined nLOT, we identified them as part of the amygdaloid BST. This radical new interpretation acknowledges a recent finding that telencephalic dopaminergic neurons project into the hypothalamus in addition to the teleostean striatopallidum (“Vd”) (Tay et al., 2011). Precommissural groups of dopaminergic neurons build the (topographically) anterior bed nucleus of the stria terminalis (BSTa) whereas supra- and postcommissural groups of dopaminergic neurons form the BSTp (Figure 2G). We also found a small number of dopaminergic neurons in the newly identified caudalmost portion of the subpallial medial amygdala (MeAv; Figure 2G) resembling the situation of its most probable mammalian homolog the mammalian MeApd. Likewise, dopaminergic neurons are also found in subdivision so the mammalian BST (Northcutt and Lonstein, 2011; Bupesh et al., 2014). Overall, the zebrafish dopaminergic BSTp is positioned in a laterally displaced angle and close to the *isl1*:GFP positive medial BST (BSTm; Figures 2F–J). Both the zebrafish CeA (=CeAa, CeAl, CeAd) and the dopaminergic BST neurons seemingly are located in the *isl1*:GFP negative and GABA-positive dorsal subpallium that corresponds to the dorsal lateral ganglionic eminence (dLGE). This interpretation is consistent with conserved regulatory gene expression patterns and developmental studies in zebrafish (Mueller et al., 2008; Mueller and Wullimann, 2009; Tay et al., 2011; Ganz et al., 2012).

## Nucleus of the Lateral Olfactory Tract (nLOT) and Integrative Olfactory Pallial Nucleus [IOP; Mammalian Lateral Pallium (LP)]

### *Tg(lhx2a:GAP-YFP)* Labels Main Olfactory Bulb Projections to Two Cortical Nuclei

Pivotal for assigning homologies in zebrafish is the correct interpretation of the posterior olfactory nucleus “Dp,” which partially forms through a peculiar radial cell migration (Mueller and Wullimann, 2009; Mueller et al., 2011). Quite a number of authors have questioned the origin of Dp, its homology to mammals, exact delineation and position (Nieuwenhuys, 2009b). To solve this debate, we re-investigated the distribution of secondary olfactory projections using the transgenic line *Tg(lhx2a:GAP-YFP).* According to the study that introduced this transgenic zebrafish line, olfactory bulb neurons allegedly send their projections to a large single olfactory receptive pallium, usually called the posterior pallial zone (Dp) (Miyasaka et al., 2009). However, our results showed that these neurons project not to one but to two separate pallial nuclei (Figures 2J,N), which in turn differ by the respective presence or absence of parvalbumin staining. The overlooked projection innervates the parvalbumin-positive integrative olfactory pallium (IOP, Figure 2N) previously mislabeled as a part of the dorsolateral pallial territory (“Dl”) in the adult zebrafish brain atlas (Wullimann et al., 1996). This parvalbumin-positive region is the putative homolog to the mammalian lateral pallium (LP) and probably best viewed as a teleostean counterpart to the mammalian entorhinal cortex, which considered a part of the hippocampal formation. In contrast, the second parvalbumin-free region, as our data indicate, represents the amygdaloid nucleus of the lateral olfactory tract (nLOT; Figure 2J). The zebrafish nLOT, like its mammalian counterpart, is a molecularly heterogeneous composite structure whose GABAergic and glutamatergic components originate from different primordial sources. For example, both the rostral- and caudalmost *vGlut2a*:GFP positive cell masses of the nLOT (Figure 2C) are most likely generated through radial migration from the *lhx5*-negative/*vGlut2a*-positive thalamic eminence (EmT; this study). In addition, other glutamatergic, *vGlut2a*-negative cells are probably derived from a region closely adjacent or overlapping with the primordial pallial amygdala (Mueller et al., 2011). In contrast, the presence of GABAergic portions of the nLOT (Figures 2O–Q) suggests a subpallial origin of these neurons. More posterior glutamatergic yet *vGlut2a*:GFP negative portions that are not innervated by secondary olfactory fibers may be derived from other telencephalic or thalamic sources. Previously, the nLOT was misinterpreted both as part of the teleostean ventral portion of the medial (hippocampal) pallium (“Dlv”) and the so-called posterior zone of the pallium (Dp) of Wullimann (Wullimann and Mueller, 2004b; Northcutt, 2008; Ganz et al., 2012). However, the absence of substance P (this study) and previously reported absence of dorsal raphe serotonergic input (Lillesaar et al., 2009) speak against higher integrative function.

## The Supra- and Postcommissural Extended Medial Amygdala Network

### The Supracommissural Extended Amygdala (CeAd, BSTm, BSTp, MeAv, pAmy)

We angled cross sections perpendicularly to the rostro-caudal axis of the zebrafish telencephalon to visualize the supracommissural BSTm/MeAv/CeAd configuration as an oval structure sandwiched between the caudalmost DM (*vGlut2a*-driven GFP positive putative pAmy) and the anterior commissure (Figures 2F–J). The relative position of these territories between both the anterior commissure (ac) and pallial-subpallial border (PSB) corresponds to the topological situation in mammals, in such a way that the pallidal BSTm is located adjacent to the ac, followed by an intermediate positioned striatal MeAv, and the CeAd being closest to the PBS. We refer to this oval composite structure as the supracommissural standard configuration, which permits an easy navigation between the supracommissural and postcommissural amygdala (Figures 2K–N). In the latter, the MeAd merges with the posteromedial pallial nucleus (PMPa) at the caudalmost telencephalic expansion (Figures 2K–N). In the former, a group of *isl1*-GFP-positive neurons contribute to the medial bed nucleus of the stria terminalis (BSTm; Figures 2F,G), whereas *isl1*:GFP-negative TH-expressing cells form the posterior division of the BST (BSTp) (Figures 2F,G). A small number of *isl1*:GFP-negative and TH-and GABA-positive neurons contributes to the MeAv. The positions of the medial amygdaloid nuclei (MeAd, MeAv, MeAp) are consistent with the topological positions of the redefined BST nuclei (BSTm, BSTa, BSTp), the nLOT, the PMPa, and the IOP. In these posterior sections, numerous calretinin-positive neurons populate all MeA- and BST nuclei, which distinguish them from CeA territories that are largely free of calretinin cells. The distribution of calretinin cells in zebrafish resemble the situation described for mammals (Wojcik et al., 2013).

### The Postcommissural Amygdala (MeAa, MeAd, MeAv, MeAp, PMPa, nLOT)

Next, we analyzed calretinin cell and fiber patterns in GABAergic subcortical amygdaloid territories (Figures 2H,I,K,M) to decipher the topological relationship between the MeA nuclei and the newly identified PMPa. We found that the PMPa shows extensive calretinin cell and fiber staining continuous with the dorsal division of the zebrafish medial amygdala (MeAd; 2I). Moreover, we define the posterior division of the MeA (=MeAp) based on the presence of a large population of otpa-positive neurons (Figure 2L). In addition, a contiguous band of calretinin-positive neurons defines both the MeAd and MeAv (Figure 2M). Connections between MeAd and PMPa are suggested by calretinin fibers that arrive via four fiber bundles in the ventralmost aspect of the MeAd (Figure 2I). In the dorsal GABAergic subcortical MeAd at posterior sections, these calretinin bundles fuse into one bilateral continuous band projecting into the PMPa (Figure 2I). The ramification patterns of these calretinin fibers suggest a functional link between MeA and PMPa. Supporting this interpretation are substance P positive fibers that emanate from the olfactory bulb and reach the medial amygdala (MeA) at posteriormost sections (Figures 4D–F,G–J). Specifically, these substance P positive fibers also innervate the zebrafish PMPa (black thick arrows in Figures 4E,F pointing within PMPa) supporting its functional relationship with the MeA. In addition, numerous calretinin positive cells in the territories of the posterior medial extended amygdala (MeAd, MeAp) support its redefined homology. We speculate that many of these calretinin positive neurons mediate odor-cued behavior and, like in mammals, emanate from hem like organizing centers including the thalamic eminence (Bielle et al., 2005; Huilgol et al., 2013; Huilgol and Tole, 2016).

### Substance P (SP) in Extended Amygdala Marks Accessory Olfactory Nuclei

In mammals, the MeA mediates reproductive behavior in response to sex-pheromones and receives projections from the accessory olfactory bulb (Abellan et al., 2013). These characteristics have been used in classical studies to define the MeA in non-mammalian vertebrates with a vomeronasal organ such as amphibians and lungfish (Moreno and González, 2003; González et al., 2010). Given that zebrafish like other ray-finned fish lack a vomeronasal organ and accessory olfactory bulbs, it is generally assumed that an accessory pathway and thus the medial amygdala is absent in zebrafish. However, the olfactory epithelium of ray-finned fish contains sensory neurons with pheromone-binding vomeronasal-like as well as pheromone-binding (non-vomeronasal) olfactory receptors critical for reproductive behaviors (Ahuja and Korsching, 2014; Behrens et al., 2014). Hence, we tested the existence of several medial extended amygdaloid territories and the PMPa also through immunohistological detection of substance P (SP) in adult zebrafish. In both amphibians and mammals this neuropeptide labels accessory olfactory bulb projections to the MeA (Moreno and González, 2003; Davidson et al., 2006). Specifically, the presence of extensive SP fibers and numerous SP positive neurons separates the mammalian MeA from the central amygdala (CeA) (Emson et al., 1978). In adult zebrafish two SP-positive bulbofugal tracts innervate the medial extended amygdaloid nuclei (Figures 4G–J) in the rostral (Figures 4A–C) and caudal (Figures 4D–F) zebrafish telencephalon. The SP-positive MeA nuclei thus can be clearly distinguished from the negative CeA (CeAa and CeAl; Figures 4B,C). Even more significant is the finding that SP fibers pass through the MeA (MeAd in Figures 4D,E) and project to the dorsalmost regions of the PMPa and the integrative olfactory pallium (IOP; Figures 4E,F). This finding supports the identification of these newly defined territories. The nLOT completely lacks substance P fibers in contrast to the IOP (Figures 4C–F). This finding is consistent with the interpretation that the zebrafish IOP integrates accessory olfactory-like and main olfactory and gustatory information. In contrast, the absence of substance P fibers in the nLOT suggests that this structure integrates main olfactory and gustatory information only.

## Tela Attachment Sites in Comparison to Radial Glia Cells and *vGlut2a*-Driven GFP Indicate Complex Telencephalon Eversion

Our molecular data and amygdala framework as outlined above is incompatible with current eversion models, especially with the idea that the zebrafish telencephalon develops through concentric growth that causes a simple “outside-in pallial” organization as proposed based on radial glia cell distribution and genetic fate mapping (Furlan et al., 2017). We postulated that the conclusion of these results are due to inaccurate anatomical analyses of both the periventricular radial glia and pial surfaces of the pallium and misleading claims of earlier studies suggesting that radial glia and tela attachment sites reflect a simple pallial eversion in teleosts (Nieuwenhuys, 2009a, b). To test this hypothesis and define periventricular sites versus respective pial sites, we mapped the tela attachment sites and distribution of radial glia somata and processes. For this purpose, we used antibodies against parvalbumin (PV), GFAP and GFP in the transgenic line *tg(vGlut2a:GFP)* allowing us to correctly annotate distribution of radial glia cells and their processes and tela attachment and fusion sites.

### The Tela Attachment and Fusion Sites Indicate Complexly Everted Pallial Organization and Contributions of the (Pre-)Thalamic Eminence (EmT)

**FIGURE 5:**
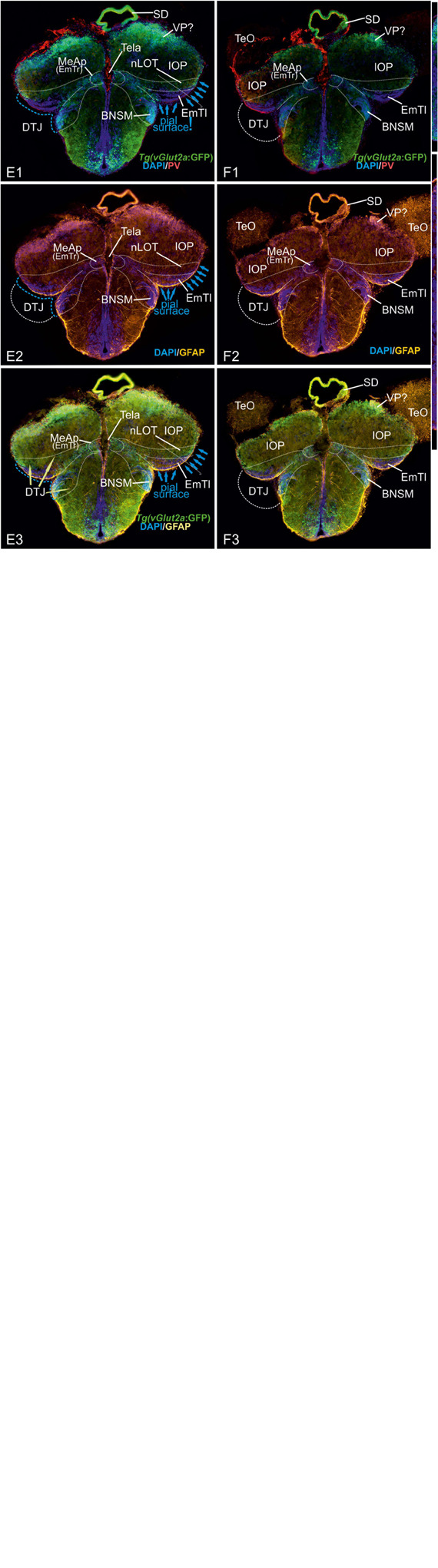
Radial glia cells and Tela Attachement Sites Confirm Medial, Dorsal, and Thalamic Eminence Identification. We performed triple fluorescence immunostains against parvalbumin (PV), GFP, and GFAP in the brains of Tg(vGlut2a:GFP) counterstained against DAPI. **(A1–C2):** A1 shows different aspects of a rostral section highlighting that one large parvalbumin- (PV-) positive tela attachment site is located at the ventricular site of the dorsal pallium and pallial amygdala (white arrows in **A1,A2,B,C1**). The tela choroidea is fused (green arrows in **A1,A2,B,C1**) with the proliferative stem cell layer of the dorsolateral zone (“Dl”) supporting its medial pallial (“hippocampal”) identity. The nLOT tract shows a characteristic indentation **(A2,C2)** and no GFAP stained cell somata at its lateralmost aspect corroborating this area as the pial, not ventricular site. Notably, all radial glia of the candidate dorsal pallium do send their processes towards the pallial-subpallial border (PBS). These radial glia are attached to the topographically ventral side of the nLOT next to those of the medial pallium. Radial glia of the nLOT are not visible in this orientation suggesting that their radial domain is different from pallial zones. This finding supports the interpretation that the vGlut2a-driven GFP domain is a derivative of the EmT. **(D1A–D2B)** At mid-telencephalic sections, both radial glia cells and tela attachments show the same distribution. At the point where proliferative zones of the DM and medial pallium (MP) meet and grow over the dorsal pallium, a reduced number of radial glia cells of the dorsal pallium are visible. Radial glia of both MP and DM cut through the territory of DP and send their processes beneath the nLOT. The probable diencephalic attachment site of the tela choroidea is located at the nLOT (gray arrows in **D1b,D2b**). Again, there are no GFAP-positive glia cell somata at the most lateral cell groups corroborating its pial nature. The nLOT in this orientation shows no radial glia processes supporting their postulated EmT origin. At caudalmost sections, the study identifies the diencephalic -telencephalic junction (DTJ) that comprises territories formerly assigned to the telencephalon such as the posterior medial amygdala (MeAp/EmTr) and the vGlut2a-driven GFP positive nLOT territory that spans a region next to the MeAp/EmTr to the lateral (pial) surface (blue arrows in E1-E3). The BNSM was formerly identified as a derivative of the EmT in zebrafish and is often mistaken as the entopeduncular nuleus proper (ENv) Mueller and Guo 2009). Note, that the tela choroidea is attached to the MeAp/EmTr indicating its periventricular site **(E1,F1)**.

We found that the PV antibody visualized the telencephalic tela choroidea (Figure 5, specifically 5A1/A2, D1-D2B, and E1/F1). Most strikingly, and in sharp contrast with former studies, we found that the tela choroidea is fused with at the periventricular site of the dorsolateral zone (“Dl”) corroborating the interpretation that this zone is the zebrafish MP (Figures 5D1B,D2B). This finding thus falsifies Nieuwenhuys hypothesis stating that the tela is attached solely to what he identified as (telencephalic) “nucleus taeniae”: (Nieuwenhuys, 2009b). Our results, in contrast, indicate that he misinterpreted what would be traditionally viewed as a diencephalic attachment site as being telencephalic. This fact becomes particularly evident at caudalmost sections in the region that we describe as *diencephalic-telencephalic junction* (DTJ) in this study (Figures 5E1–F3). Within the DTJ, the zebrafish *vGlut2a*-driven GFP positive (*lhx5-*driven GFP negative) thalamic eminence (EmT) is contiguous with a portion of the regions of the dorsoposterior zone (DP) within a transitional zone between EmT, nLOT, and IOP (*vGlut2a*-driven GFP of the nLOT as visualized in Figures 5E1–F3). The putative GFP-positive EmT-derivative (EmT-d) extends to the pial surface of the pallium occupying a small portion of the part that is usually considered Dp or Dlp. Topographically beneath this GFP positive EmT-d, we identify the lateral thalamic eminence (EmTl; Figures 5E1–F3), which in mammals is an important signaling center that contributes to the development of the nLOT (Remedios et al., 2007; Huilgol and Tole, 2016; Ruiz-Reig et al., 2017). The EmTl is most pronounced and appears as a pseudo-layered or folded structure at the DTJ probably due to bending processes during development. The characteristic distribution of the *vGlut2a*-driven GFP neurons close to the pial surface of the pallium, the lateral displacement of the nLOT in connection with an indentation of the outer pallial rim, as well as the close relationship with the diencephalic tela attachment site together suggest that the *vGlut2a*-driven GFP positive portions of the nLOT represent derivatives of the thalamic eminence (EmT-d) as summarized in the schematic Figure 8). We postulate that *vGlut2a*-driven GFP positive cell masses of the nLOT in its most rostral (e.g., Figures 2A–C, 5A1–C2, D1B) and caudal aspects (are derived from the *vGlut2a*-driven portions of the EmT. Again, what is visible as a tela attachment site across the entire length of the nLOT represent a diencephalic attachment site (Figures 5D1B,D2B), not homologous to the one that defines the medial pallium as proposed before.

This drastic reinterpretation of the tela attachment sites as well telencephalic homologies to mammals also concerns an important structure at the DTJ, which we called the posterior medial amygdala (MeAp). Notably, we found that PV also stains the tela attachment site of what we identified as the rostral portion of the thalamic eminence (EmTr) and which we consider a part of the medial amygdala and consequently called it the *posterior medial amygdala* (MeAp/EmTr). The MeA/EmTr has been previously proposed as being the MeA and labeled as the intermediate subpallial nucleus (“Vi”) that contains tangentially migrated hypothalamic otp-a positive neurons (Biechl et al., 2017). The facts that the tela choroidea is attached to this nucleus (Figures 5E1,F1) and that glutamatergic neurons populate this nucleus (Figures 5E1,F1) strongly suggest that this territory is an EmT derivative that forms a smaller part of the medial extended amygdala as visualized in schematic Figure 8.

### Radial Glia Somata and Processes Support Complexly Everted Pallium, Contributions of the (Pre-)Thalamic Eminence (EmT), and Presence of the Dorsal Pallium

At rostral most sections (Figures 5A1–C1), distribution and orientation of radial glia and their processes were consistent with our delineation of both the pallial amygdala (DM) and dorsal pallium (DP). The GFAP-positive radial glia of both these territories send their processes toward the subpallium and end just at the pallial-subpallial border (PBS) that is located topographically below the GFP-positive and PV-negative nLOT. However, the somewhat laterally displaced nLOT does not show radial glia somata at its lateral superficial expansion demonstrating that this part does not contain proliferative stem cells as shown before based on proliferation patterns (Wullimann and Mueller, 2004b; Lillesaar et al., 2009; Mueller and Wullimann, 2009; Mueller et al., 2011). Thus, our results again confirm that the nLOT is located at the pial surface and not at the periventricular site as suggested in simple eversion models. This finding is consistent with the interpretation that heterogeneous migrated cell masses form the nLOT. This hypothesis is strongly supported by the somewhat lateral displacement of the nLOT which leads to an indentation between nLOT and DP or MP (Figures 5A1–C2). Likewise, the overall orientation and distribution of radial glia cells supports the interpretation that the centralized zone of the zebrafish pallium (“Dc”) represents the dorsal pallium (DP) which extends into the topographical rostral most sector and includes its own germinative zone of origin (Mueller et al., 2011).

At mid-telencephalic sections (Figures 5D1A,D2B), the processes of the radial glia of both the DM and the MP cut through the DP territory and end just below the nLOT at the PSB. Interestingly, there are no radial glia cell somata nor radial glia processes visible at the lateral margins of the nLOT toward the indentation close to the tela attachement site. This finding again is consistent with our hypothesis that the *vGLut2a* positive portions of the nLOT originate from the EmTl (see schematic in Figure 5). Moreover, both the DM and the MP have overgrown the DP for the largest part, only few radial glia cells belong to DP (arrow pointing on radial glia cells of the dorsal pallium in Figures 5D1B,D2B). This finding suggests that once overgrown by DM and MP, the radial glia of DP will be replaced by those of the DM and MP.

At caudalmost sections, the processes of the radial glia located within the DM, PMPa, and IOP, again, dock onto the area topographically below the nLOT. As expected, none of the *vGlut2a*-driven GFP positive territories defined in this study as nLOT show radial glia somata at their pial site, nor do they show pronounced radial glia processes indicating that these territories have formed either through early radial migration (within a thalamic territory (EmT) or through tangential migration (late developmental stages from thalamic EmT into telencephalon). Note, that we view the *vGlut2a*-driven GFP positive portions of the nLOT as a radial domain of the EmTm or EmTl not described for earlier stages in zebrafish (Wullimann and Mueller, 2004a; Turner et al., 2016).

## Differential Expression of *vGlut2*- and *lhx5*-Driven GFP Define Subdivisions of the Thalamic Eminence at the Diencephalic-Telencephalic Junction

**FIGURE 6:**
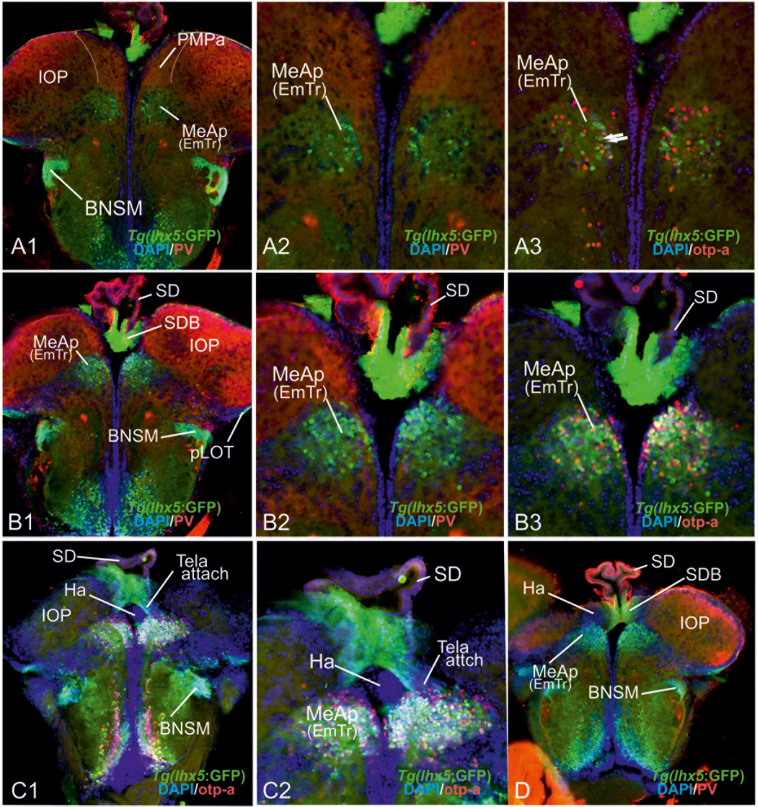
The posterior medial amygdala (MeAp) is identical with the rostral thalamic eminence (EmTr). **(A1–D)**: The comparison between lhx5-driven GFP, parvalbumin (PV), and otp-a protein distribution shows that the lhx5-driven GFP positive EmTr reaches from its periventricular site (indicated by tela choroidea attachment in **C**). Note, the lhx5-driven GFP positive base (SDB) of the saccus dorsalis (SD) connects to both the Ha (in C1/2 and D) and the MeAp/EmTr (best visible in C1/2)hh. Like in mice, the posterior territory of the lateral olfactory tract seemingly does express lhx5 as indicated by GFP expression in Tg(lhx5:GFP) **(A1,B1,C1,D)**.

Comparing *lhx5*-driven GFP versus distributions of otp-a and *vGlut2a*-driven GFP revealed the detailed architecture of the adult zebrafish EmT and its topological relationships to both the EmTr/MeAp (former “Vi”) and nLOT (Figures 6A1–D). Specifically, the results showed that otp-a protein distribution strongly overlaps with *lhx5*-driven GFP positive neurons within the EmTr/MeAp where the tela choroidea attaches at posteriomost sections close to the border to the habenula which is separated from the EmTr/MeAp by the base of the saccus dorsalis (SDB; Figures 6C1,C2,D). The *lhx5*-driven GFP expressing neurons at the posteriormost section reach to the periventricular site indicating that this proliferation zone gives rise to the *lhx5*-driven GFP positive cells, some of which are double-positive for both otp-a and *lhx5*-driven GFP (Figures 6A3,B3). This finding suggests that at least a fraction of these otp-a positive neurons is derived from the EmTr.

## Discussion

Teleost fish with more than 26,000 species comprise the largest vertebrate group and exhibit sophisticated cognitive capabilities underlying social and reproductive behaviors (Fernald, 2017). Yet to this day, the neural basis of these complex behavioral repertoires has remained elusive. Specifically, the field lacks a precise definition of the teleostean amygdala, whose counterparts in mammals form the regulatory core of the emotional brain essential to emotion, cognition, and social behavior (Northcutt, 2008). The mammalian amygdala shares many characteristics with other tetrapods and their sister group, the lungfish, which suggests that a complex tetrapod-like amygdala ground plan originated in the common ancestor of lungfish and tetrapods (Moreno and González, 2007; González et al., 2010; Abellan et al., 2013). It is currently, however, not understood how the amygdala is organized in more basally derived fish. In teleosts, eversion of the forebrain made direct comparisons difficult with tetrapod forebrains that develop through evagination (Figure 1; Nieuwenhuys, 2009b). We choose to focus and decipher the teleostean amygdala ground plan in zebrafish, because it represents an important model system for brain development and disease. What is more, earlier studies on zebrafish provide rich molecular information regarding pallial and subpallial entities including putative homologs of the mammalian iscortex and hippocampus (Mueller and Guo, 2009; Mueller et al., 2011; Ganz et al., 2014). Key processes, moreover, of the telencephalic eversion have been uncovered in zebrafish, providing an ideal foundation for our redefinition of the zebrafish amygdala (Mueller and Wullimann, 2009; Mueller et al., 2011; Folgueira et al., 2012).

Combining molecular characterizations with evolutionary and developmental considerations, we describe the entire zebrafish amygdala and its relationships with pallial, subpallial, and EmT territories. Our results stress that a dense almost indivisible continuum of thirteen territories forms the zebrafish amygdala. For the first time, we identify the nLOT and the posteromedial pallial nucleus (PMPa), both of which are integral elements of the zebrafish amygdala and the primary olfactory pallium. In addition, we show that the highest integrative olfactory zone (“Dp proper”) occupies a sector that was previously considered a posterior portion of the dorsolateral pallium (“Dl” or “Dlp”). To avoid future misunderstandings, we call this region the integrative olfactory pallium (IOP), which we postulate represents the homolog of the lateral pallium (LP; mammalian entorhinal cortex). Despite their everted positions, each pallial amygdaloid territory is defined in this new framework through its conserved relationships with subpallial nuclei.

### Neural Systems Organization of the Zebrafish Amygdala in Relation to Olfaction

The zebrafish amygdala consists of three functional networks similar to the situation described for mammals: (1) the main olfactory system, (2) the accessory olfactory-like or reproductive amygdala, and (3) the central and pallial amygdala (CeA-pAmy) network (mammalian BLA) regulating autonomic functions and mediating associative emotional learning. In other words, (1) the nLOT and two related nuclei of the bed nucleus of the stria terminalis (BSTa, BSTp) are part of the zebrafish main olfactory network. (2) The zebrafish accessory olfactory-like pathway consists of the PMPa, four medial amygdaloid territories (MeAa, MeAd, MeAv, MeAp/EmTr), and the medial BST (BSTm). The MeAp/EmTr, previously termed intermediate subpallial nucleus (Vi), has already been identified as an olfactory subpallial nucleus (the “MeA”) that responds to socially relevant olfactory information (Biechl et al., 2017). (3) The zebrafish central amygdala (CeA = CeAa+CeAl+CeAd) sits close to and topographically below the region that is commonly viewed as the teleostean pAmy (Portavella et al., 2004a, b; Wullimann and Mueller, 2004b; Martin et al., 2011; von Trotha et al., 2014; Ruhl et al., 2015).

This separation of the three neural systems becomes visible when looking at the molecular characteristics that define mature neurons. For example, the branching formations of both substance P and calretinin fibers reflect the topological relationship and connectivity between newly identified core territories of the extended medial amygdala (MeAd, MeAv, MeAp). As a unifying characteristic, these accessory olfactory-like territories share the presence of numerous calretinin-positive neurons. This fact clearly separates them from anterior central amygdaloid territories that lack such calretinin neurons. Likewise, among central amygdaloid territories, the CeAd stands out as a laterally displaced sector enriched with parvalbumin fibers and sparse parvalbumin-neurons. Similarly, amygdaloid structures of the main olfactory-like pathway, such as the nLOT and the dopaminergic BST territories, lack substance P fibers.

Taken together, our new amygdala framework reveals an intricate zebrafish pallial-subpallial amygdala network of heterogonous molecular structure that resembles the amygdala of mammals with prominent olfaction, such as macrosmatic rodents.

Hallmarks of our new amygdala paradigm are the identification and molecular definition of the EmT territories (EmTr; EmTl, EmTm), the nLOT, and the highest olfactory pallial zone, which we termed the integrative olfactory pallium (IOP). Their precise demarcation, chemoarchitecture and topology allowed us to clarify previously mislabeled neighboring structures (see Supplementary Table S1 that lists labeling inconsistencies across teleosts). The zebrafish IOP, for example, was previously interpreted as part of the dorsolateral (Dl) pallial zone by Wullimann et al. (1996). As such, it was incorrectly considered as a non-olfactory extension of the teleostean medial pallium (mammalian hippocampus). Our identification of the actual Dp region—as integrative olfactory pallium (IOP) in its redefined position in the dorsoposterior pallium—posits also a topological correspondence to the mammalian entorhinal cortex (=lateral pallium; LP). That is, we postulate that the IOP/Dp proper is not part of the “teleostean hippocampus” (“Dl”) but like the mammalian entorhinal cortex an important building block of the “teleostean hippocampal formation-like system.” In other words, we suspect that the IOP is critical for both olfactory integration and olfactory-related navigation similar to the mammalian entorhinal cortex. Also, the zebrafish nLOT was previously misinterpreted either as part of the “teleostean hippocampus” (“Dlv”) or the posterior olfactory pallium (“Dp”) (Wullimann et al., 1996; Ganz et al., 2014). Confusion about Dp has also led to functional misinterpretations and the erroneous assumption that the Dp territory represents the highest integrative olfactory pallial zone (Jacobson et al., 2018). Our identification of the zebrafish nLOT as a potentially lower integrative structure emphasizes deeply conserved evolutionary links between the teleostean and mammalian amygdala and primary olfactory pallium. The identification of various EmT territories and their structural relationship to the zebrafish amygdala clarifies the unusual (compared to non-actinopterygian vertebrates) everted topology of the teleostean forebrain that has been subject of debate among comparative neurologists for over a century (Nieuwenhuys, 1963, 2009b; Wullimann and Mueller, 2004b; Yamamoto et al., 2007; Northcutt, 2008; Mueller and Wullimann, 2009).

Our data also indicate deep and unexpected relationships between the DM, the PMPa, the integrative olfactory pallium (IOP), and the nLOT. For example, we show that the PMPa needs to be viewed as a pallial passage functionally linked to the extended medial amygdala and cannot simply be described as an extension of DM (anterior Dm) (Northcutt, 2008; von Trotha et al., 2014; Ruhl et al., 2015). Critically positioned between the DM (important for motivational states), the IOP and the extended medial amygdala (processing pheromones and social cues), the PMPa, we postulate, integrates accessory olfactory information and motivational states. In contrast, the newly identified dopaminergic BST nuclei (BSTa and BSTpd) form a functional relationship with the nLOT. As a part of the main olfactory system, the BSTa and BSTpd most likely modulate the activity of the nLOT. We postulate that the nLOT itself integrates main olfactory and gustatory information, given that this structure receives projections from both systems (Vaz et al., 2017; Yanez et al., 2017).

### Molecular Amygdala Characteristics and Functional Organization

Our drastically revised zebrafish amygdala-olfactory systems paradigm is also supported by previous findings about the expression patterns of conserved gene regulatory genes and the development of the zebrafish telencephalon. The interpretation of the *isl1*:GFP negative BSTa; BSTp, CeA, MeAd as mainly dLGE-derived territories is compatible with conserved regulatory gene expression patterns and developmental studies of the zebrafish telencephalon (Costagli et al., 2002; Mueller et al., 2008; Mueller and Guo, 2009; Ganz et al., 2012; Folgueira et al., 2012). A study has shown as well that the adult *isl1*-driven GFP expression strongly resembles the larval expression (Baeuml et al., 2019). This fact supports our interpretation that the isl1-free GABAergic zone in fact represents an evolutionarily expanded dLGE territory. However, we cannot exclude the possible presence of pallidal and striatal neurons that may have migrated from the *isl1*-positive territory and subsequently suppressed *isl1*-expression. We suspect also that amygdaloid territories might be even more heterogeneous than this study proposes. For example, the zebrafish BST may include a territory with isl1-negative and nkx2.1 positive neurons as has already been suggested (Ganz et al., 2012).

Equally important for understanding the ground plan is that the distribution of mature neuronal phenotypes conforms with the function of these amygdaloid territories. For example, even though the overlapping distribution of *vGlut2a*-driven GFP (this study) and *emx3* mRNA (Ganz et al.) render the zebrafish DM a relatively homogenous histogenetic unit, the region most likely subdivides into functionally distinct sectors. A recent study showed that emx3-positive glutamatergic subpopulations, called “Dm120A,” innervate zebrafish hypothalamic territories (Lal et al., 2018)(Ganz et al., 2014). In contrast, the mammalian central amygdala projects to the hypothalamus via GABAergic neurons. Here the zebrafish pallial amygdala candidate (DM) seems to deviate, at least partially, from the mammalian one. Mammals except marsupials also do not possess an emx3 ortholog (Kuraku, 2010) and instead express Emx1 in specific nuclei of the pallial amygdala (Medina et al., 2017). In fact, the mammalian basolateral amygdala (BLA)—critical for associative emotional learning and in all likelihood the most comparable region to the zebrafish DM region – contains Emx1 positive neurons (Cocas et al., 2009) (Medina et al., 2017). Other studies also support this comparability between mammalian BLA and part of the zebrafish DM. In particular the significant expression of cannabinoid receptor 1 in the zebrafish DM (Lam et al., 2006), is also a key characteristic of the mammalian BLA (Katona et al., 2001). These findings point to deeply conserved neurophysiological and functional parallelisms between ray-finned fish and tetrapods. Previous studies showed also that the zebrafish DM is involved in associative learning and endocannabinoid signaling critical for its function (von Trotha et al., 2014; Lau et al., 2011; Ruhl et al., 2015, 2017). As a result, the zebrafish amygdala needs to be understood as a mosaic of conserved and acquired characteristics much like other parts of the zebrafish brain (Costagli et al., 2002; Wullimann and Mueller, 2004b; Adolf et al., 2006; Northcutt, 2008; Mueller, 2012).

### Zebrafish Eversion and Amygdala – Complex and Constrained

What is more, our data drastically extend a complex telencephalon eversion model characterized by a centralized isocortex-homolog (dorsal pallium) and a migratory stream across the pallium only in an earlier study (Mueller et al., 2011). Our identification here of several previously overlooked structures, including the principal olfactory pallium (IOP), the nLOT, and a set of EmT territories alongside documentation of radial glia distribution and attachment sites of the tela choroidea, paint a new and complex picture of the zebrafish eversion. Our results indicate, that a complete and complex outward-growing (eversion) process rearranges the zebrafish forebrain constrained by the thalamic eminence (EmT), the organizing center sitting at the diencephalic-telencephalic junction (DTJ, Figure 8). The presence of the thalamic eminence in the newly demarcated diencephalic-telencephalic junction (DTJ) of adult zebrafish supports a highly regulated outward-growing process that starts during early development. This hypothesis is consistent with embryonic eversion processes as well (Folgueira et al., 2012). During larval and juvenile stages, the eversion gains in complexity through radial migratory processes and the overgrowing of the dorsal pallium by both the DM (putative pallial amygdala) and the MP (Mueller et al., 2008, 2011; Mueller, 2012).

**FIGURE 7:**
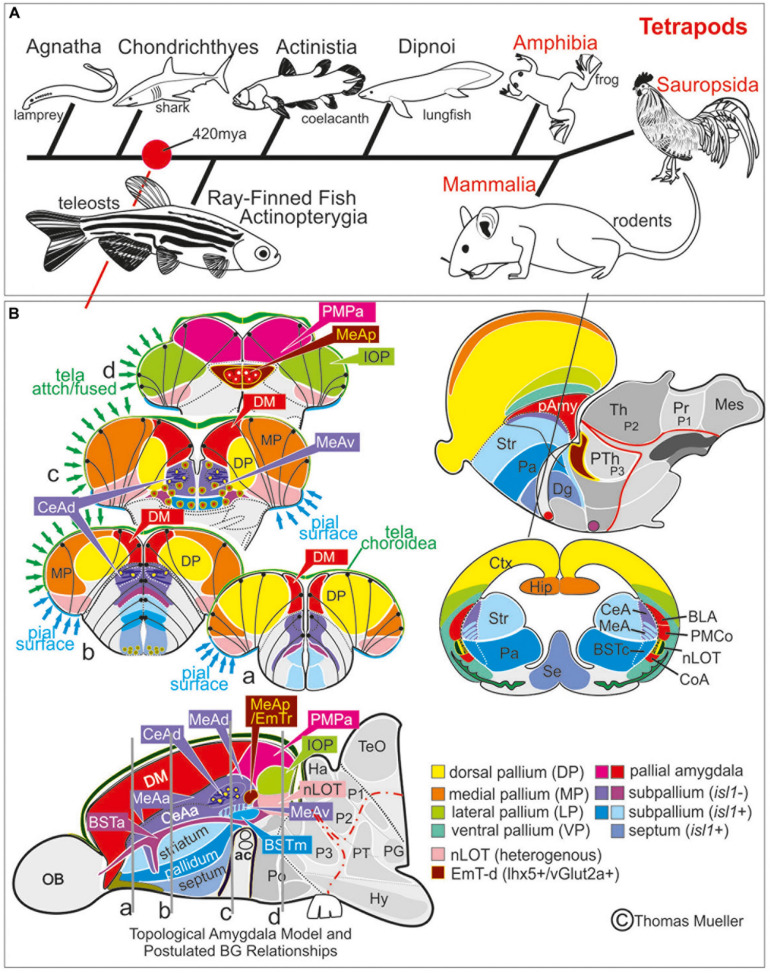
Comparison Zebrafish – Macrosmatic Rodent Amygdala. **(A)** Cladogram indicating presence of a complex amygdala ground plan and nucleus of the lateral olfactory tract (nLOT) in the last common ancestor of teleosts and mammals. Our results indicate that the last common ancestor between ray-finned fish and mammals already showed a tetrapod-like main extended amygdala ground plan (red circle). Previously, most scientists assumed that a bipartite main versus olfactory extended amygdala evolved with a vomeronasal epithelium in the last common ancestor of lungfish and tetrapods (González et al., 2010). However, molecular evidence hint toward the presence of a bipartite olfactory system already in agnathan lamprey (Chang et al., 2013). We speculate, therefore, that a bipartite and complex amygdala may evolved with the earliest vertebrates. **(B)** Prosomeric comparisons of the zebrafish/teleostean amygdala (left) with the situation in macrosmatic rodents (right). Both the schematized zebrafish brain cross sections and the parasagittal view on the left side indicate that the zebrafish amygdala holds several previously misinterpreted territories such as the bed nucleus of the stria terminals (BST), the medial amygdala (MeA) and its anterior (MeAa), posterior (MeAv) and dorsal division (MeAd), the posteriormedial pallial amygdala (PMPa), the integrative olfactory pallium (IOP), and the nucleus of the lateral olfactory tract (nLOT). Due to the teleostean-specific outward growing process (eversion), these territories lie on top of the telencephalon and cover the zebrafish homolog to the mammalian isocortex. In mammals (right side), we find the opposite situation. Here, the amygdaloid complex is located in the anterior ventral depth of the brain covered by the enlarged isocortex (modified after Mueller et al., 2011; Mueller, 2012). For molecular definition of pallial, subpallial, and EmT-derived (EmT-d) amygdaloid territories see Table 1. Abbreviations see table.

**FIGURE 8:**
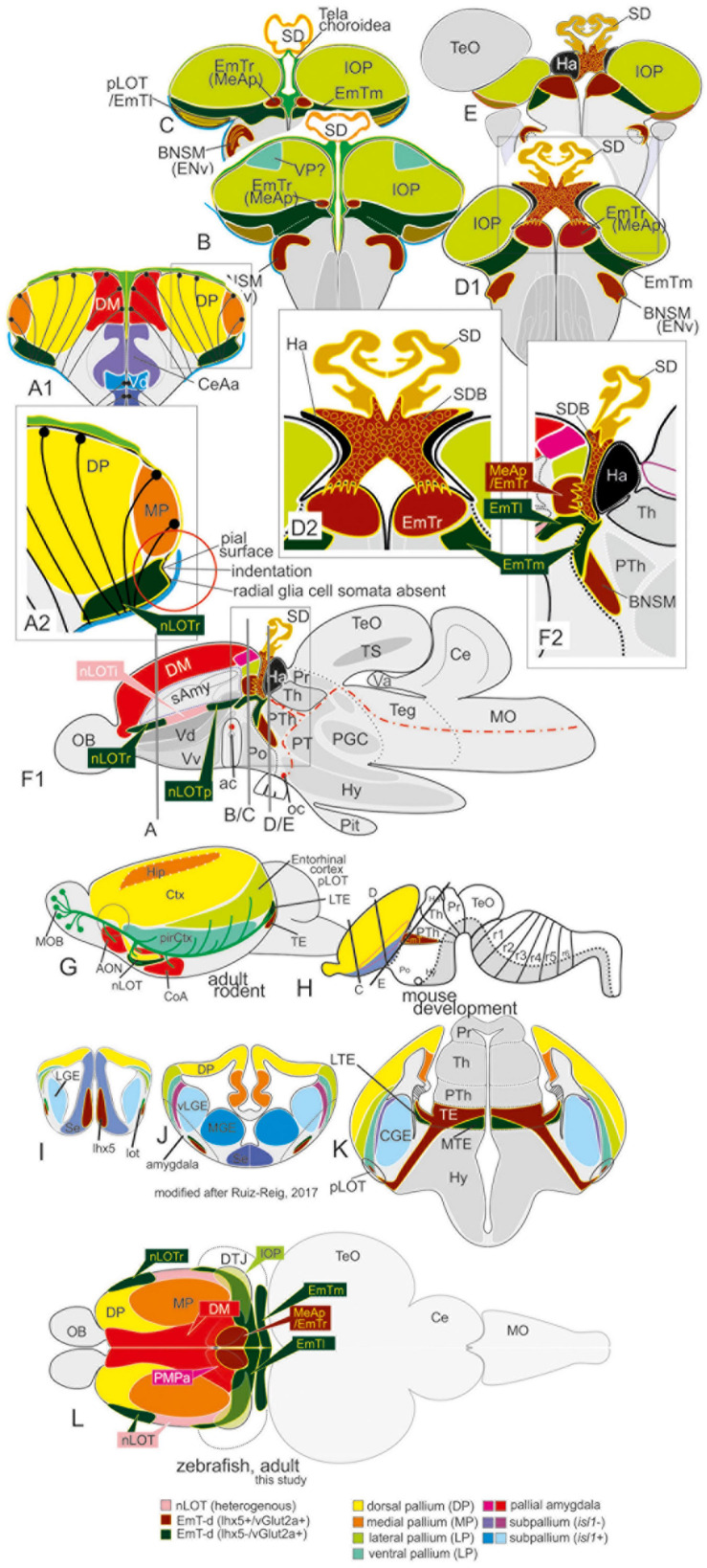
Summary schematic explaining how the zebrafish thalamiceminence (EmT) reveals ancestral topological relationships to posterior medial amygdala and olfactory pallium. **(A–F2)** Comparing topographically rostral and posterior ends of the telencephalon in transgenic lines *Tg(vGlut2a:GFP)* and *Tg(lhx5:GFP)* with distribution of parvalbumin, otp-a, and GFAP indicated a complex EmT. In sites classically defined as “telencephalic,” we identified the rostral (EmTr = *vGlut2a*+, *lhx5*+, otp-a+) and lateral (EmTl) *vlgut2a*+;*lhx5*-). At diencephalic side of the diencephalic-telencephalic junction, the EmTm can be easily identified based on *vGlut2a*-driven GFP expression and absence of *lhx5*-driven GFP, whereas the otp-a negative bed nucleus of the stria medullaris (BNSM) shows both *vlgut2a*- and *lhx5*-driven GFP. Note, that we consider the rostral and posteriormost parts of the newly defined nLOT a derivative of the EmT consistent with the absence of radial glia somata and processes in their most lateral (pial) aspects. **(G–L)** Schematics comparing brain of adult zebrafish (dorsal view) with adult rodent (idealized side view), and developmental stage to determine various EmT derivatives. The study identifies topographically rostral and posterior EmT territories based on differential expression of *vGluta*- and *lhx5*-driven GFP in comparison to otp-a expression. The dorsal view on the zebrafish telencephalon highlights “diencephalic” medial (EmTm) versus “telencephalic” rostral (EmTr) and lateral (EmTl) at the diencephalic-telencephalic junction (DTJ). The distribution of *vGlut2a*-driven GFP and *lhx5*-driven GFP in adult zebrafish strikingly resembles the situation shown in mammals (mouse) (Ruiz-Reig et al., 2017). Our complexly everted telencephalon model considers both rostral (nLOTr) and posterior portions of the most ventro-lateral expression domains of *vGlut2a*-driven GFP as radial extensions of the EmTl. The integrative olfactory pallium (IOP) is most likely a derivative of the newly defined lateral pallium of the prosomeric model and thus homologous to the entorhinal cortex.

### The Evolution of Cognition and Emotion

For the first time, our study identifies the DTJ with the EmT derivatives and their relationship to the amygdala thereby solving the zebrafish eversion for teleost ray-finned fish (actinopterygians) at large. What this study makes particularly clear is that the zebrafish telencephalon resembles the mammalian forebrain even though the teleostean everted morphology causes a markedly difference in appearance. Our findings show as well that the here newly identified EmT territories represent the missing link between the everted zebrafish forebrain and the evaginated telencephalon of mammals. Like in mammals, zebrafish EmT territories form highly conserved and intertwined forebrain elements, a fact previously overlooked causing misinterpretations of the forebrain evolution in ray-finned fish. This study also validates the prior discovery of the zebrafish dorsal pallium (mammalian iscortex), which already suggests a conserved forebrain ground plan between teleosts and tetrapods (Mueller et al., 2011). The presence of both a considerable dorsal pallium and a complex zebrafish amygdala, of course, is pivotal for the development of phenomenal consciousness, emotional sentience, and the perception of pain (Key, 2015; Sneddon, 2019). This study, therefore, stresses the need to discuss the presence and implications of emotion in fish.

Important for a better understanding of the early evolution of vertebrate amygdala and isocortex-homolog will be the identification of EmT territories in more basally derived fish. The probably highly conserved territories in actinopterygian and non-actinopterygian fish, such as agnathans (lamprey) and chondrichthyes (sharks and manta rays), might allow to solve disagreements about their forebrain evolution (Puelles, 2001; Pombal et al., 2009; Mueller, 2011; Northcutt, 2011; Schluessel, 2015; Docampo-Seara et al., 2018). Similarly, the discovery of EmT and nLOT enabled in this study to effectively compare the everted zebrafish with the evaginated forebrain of mammals. Notably, the mammalian amygdala is evolutionarily not only related to EmT and nLOT but shows also strong reciprocal connections with the prefrontal cortex, critical for cognition and goal-directed behaviors. Currently, most comparative neurobiologists assume that the prefrontal cortex, the highest integrative cortical center, represents a distinguishing characteristic of the mammalian isocortex, most pronounced in humans. However, based on the prefrontal cortex’s connections to the amygdala in mammals and conservatisms in the zebrafish amygdala, we postulate that basic amygdala-prefrontal cortex-like circuits exist in teleost fish, too. In this context, our study provides role model and rationale for the neural substrates of cognition and emotion in fishes or basally derived vertebrates. The predictable presence of amygdala-prefrontal cortex-like circuits in teleosts may eventually allow identifying the neural basis of emotional sentience in the important genetic model zebrafish.

## Data Availability Statement

All datasets generated for this study are included in the article/Supplementary Material.

## Ethics Statement

The animal study was reviewed and approved by the IACUC Committee Kansas State University, University Research Compliance Office, Manhattan, KS, United States.

## Author Contributions

TM planned the study and designed the experiments, wrote the manuscript, and carried out a portion of the immunohistological procedures and imaging. BP conducted immunhistological stainings, imaging, and statistical analyses. Both authors discussed the results and scientific interpretations.

## Conflict of Interest

The authors declare that the research was conducted in the absence of any commercial or financial relationships that could be construed as a potential conflict of interest.

## Supplementary Material

FIGURE S1 substance P fiber tracts from the olfactory bulb into the telencephalon. **(A–D)** The antibody against substance P labeled fiber bundles originating in the olfactory bulb and projecting to all extended medial amygdala (MeA) territories, posteromedial pallial amygdala (PMPa), and integrative olfactory pallium (IOP). The bulbo-telencephalo projections form *two solid tracts*; one smaller dorsally located one and a larger ventral one, plus a diffuse tract less visible in sagittal sections. The ventral substance P positive tract most likely corresponds to the lateral medial olfactory tract of cyprinids conveying pheromone information. The dorsal ascending substance P tract extends along the pallial-subpallial border (PSB) and most likely represents a derivative of the septum. Note, the nLOT, as defined in this study, lacks substance P fibers.

FIGURE S2 Sagittal brain section stained against GFAP (red), *vGlut2a*-driven GFP (green), and parvalbumin (yellow). **(A1,A2)** The distribution of *vGlut2a*-driven GFP shows the extent of the pallial amygdala DM in relation to the dorsal pallium (DP) and integrative olfactory pallium (IOP) both of which lack *vGlut2a*-driven GFP at large. The tela choroidea is closely attached (white arrows) to the DM an extents up to the olfactory bulb. Also, note that dense population of GFAP-positive fibers surround the anterior commissure similar to the situation in mammals.

TABLE S1 Nomenclature for teleostean telencephalic territories and examples for former labeling inconsistencies. ^∗^None of the former studies did establish molecularly defined histogenetic units as this study does making it impossible to perfectly compare conflicting interpretations. ^∗∗^After Biechl and colleagues (Biechl et al., 2017).

## Abbreviations

A: amygdala
ac: anterior commissure
BG: basal ganglia
BNSM: bed nucleus of the stria medullaris
BST: bed nucleus of the stria terminalis
BSTa: anterior division of BST
BSTc: central division of BST (mouse)
BSTm: medial division of BST
BSTp: posterior division of BST
Cantd, anterior commissure: pars dorsalis
Cantv, anterior commissure: pars ventralis
CeA: central amygdala
CeAa: anterior division of CeA
CeAl: lateral (migrated) division of CeA
CeAd: dorsal division of CeA
CGE: caudal ganglionic eminence
CoA: cortical amygdala (mouse)
CR: calretinin
D: dorsal telencephalon (=pallium)
Dc: central zone of D
Dd: dorsal zone of D
Dg: diagonal domain
Dl: lateral zone of D
dLGE: dorsal LGE
Dlv: ventral subdivision of Dl
Dlp: posterior territory of Dl
Dm: medial zone of D
DM: precomissural (vGlut2a positive) territory of Dm
DP: dorsal pallium
Dta: diencephalic tela attachment site
Dp: posterior zone of D
DTJ: diencephalic-telencephalic junction
EmT: thalamic eminence
EmT-d: EmT-derivative
EmTl: lateral EmT
EmTm: medial EmT
ENv, entopeduncular nucleus: ventral part
Fr: fasciculus retroflexus
Hy: hypothalamus
LGE: lateral ganglionic eminence
LP: lateral pallium
LTE: lateral thalamic eminence (mouse)
MeA: medial amygdala
MeAa: anterior division of MeA
MeAd: dorsal division of MeA
MeAp: posterior division of MeA (zebrafish)
MeApd: posterior division of MeA (mouse)
MeAv: ventral division of MeA (zebrafish pendant to mouse MeApd)
MP: medial pallium
MTE: medial thalamic eminence (mouse)
nLOT: nucleus of the lateral olfactory tract
nLOTi (zebrafish): intermediate (vGlut2a-neg.) nLOT
nLOTp (zebrafish): posterior (*vGlut2a*-pos.) part of nucleus of the lateral olfactory tract
nLOTr (zebrafish): rostral (vGlut2a-pos.) nLOT
oc: optic commissure
PA: pallidum
pAmy: pallial amygdala
PGC: preglomerular complex
pirCtx: piriform cortex
Pit: pituitary
pLOT: posterior territory of lateral olfactory tract
PMCo: (mammalian) posteromedial cortical amygdala
PMPa: posteromedial pallial nucleus
PPa, parvocellular preoptic nucleus: anterior part
Pr: pretectum
PT: posterior tuberculum
PTh: prethalamus
sAmy: subpallial amygdala
PV: parvalbumin
SD: saccus dorsalis
SDB: base of SD
Se: septum
Str: striatum
tela attch: attachment point of tela choroidea
TE: thalamic eminence (mouse)
Th: thalamus
TH: tyrosine hydroxylase
TS: torus semicircularis
V: ventral telencephalon (=subpallium)
Vd: dorsal zone of V
Vdd: dorsalmost territory of V (=“extended dLGE”)
Vi: intermediate zone of V
Vl: lateral zone of V
vLGE: ventral LGE
VP: ventral pallium
Vv: ventral zone of V.
