# Supplementary figures and images for: The Zebrafish Amygdaloid Complex – Functional Ground Plan, Molecular Delineation, and Everted Topology
Source: Front Neurosci. 2020 Jul 16;14:608. doi: 10.3389/fnins.2020.00608 (PMC7378821; doi:10.3389/fnins.2020.00608)

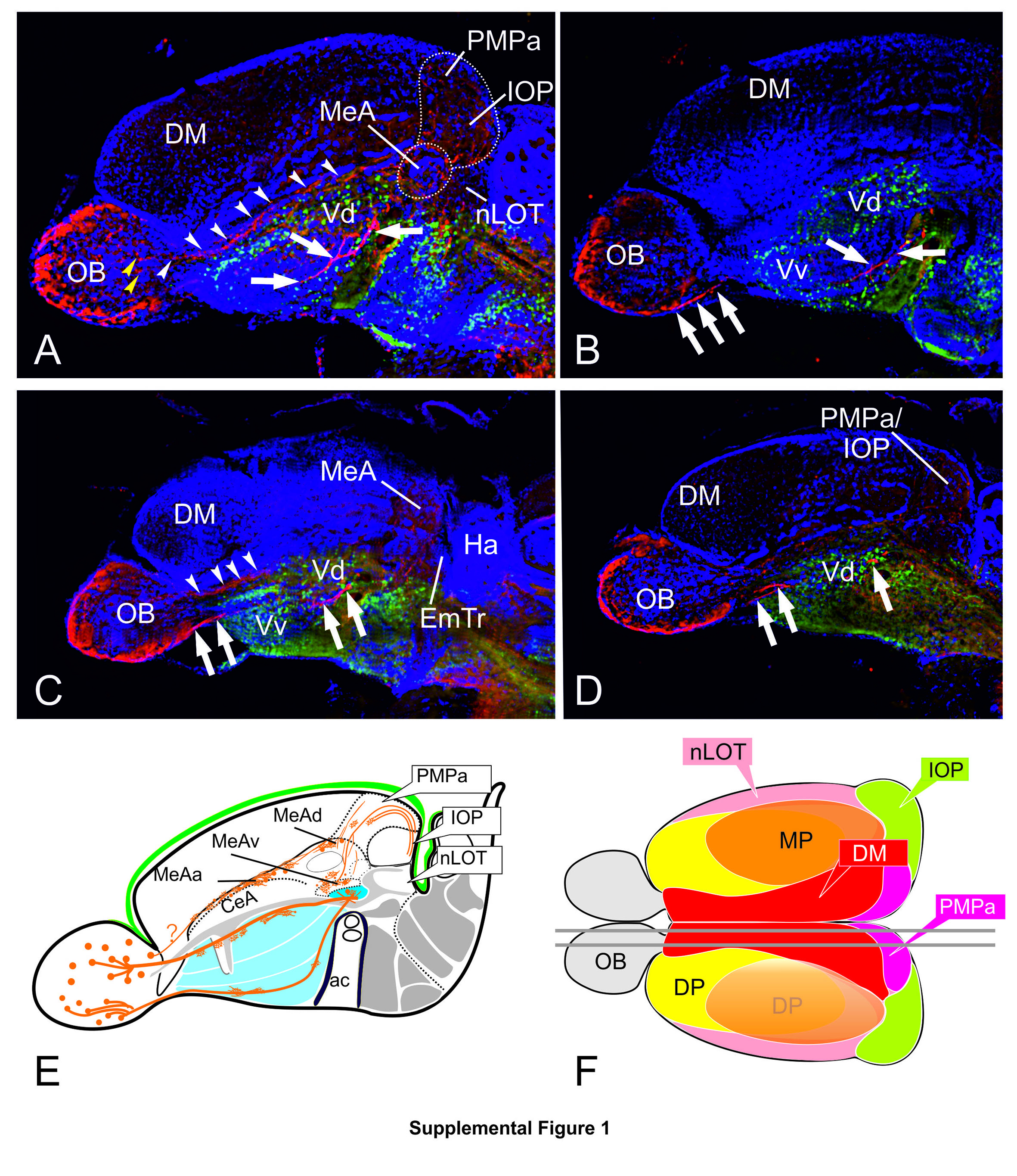

Supplement: FIGURE S1 — substance P fiber tracts from the olfactory bulb into the telencephalon. (A–D) The antibody against substance P labeled fiber bundles originating in the olfactory bulb and projecting to all extended medial amygdala (MeA) territories, posteromedial pallial amygdala (PMPa), and integrative olfactory pallium (IOP). The bulbo-telencephalo projections form two solid tracts; one smaller dorsally located one and a larger ventral one, plus a diffuse tract less visible in sagittal sections. The ventral substance P positive tract most likely corresponds to the lateral medial olfactory tract of cyprinids conveying pheromone information. The dorsal ascending substance P tract extends along the pallial-subpallial border (PSB) and most likely represents a derivative of the septum. Note, the nLOT, as defined in this study, lacks substance P fibers. [file Image_1.JPEG]

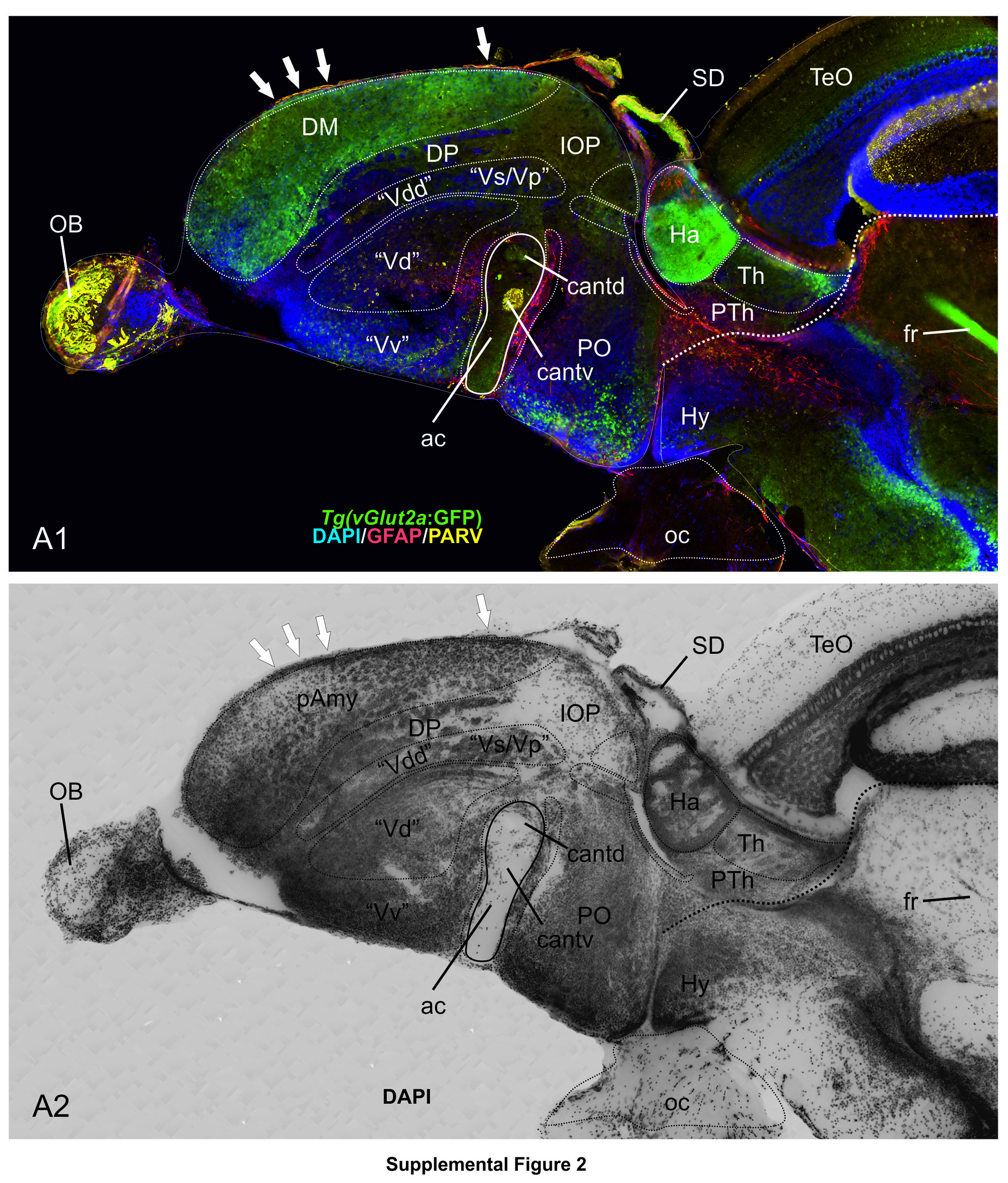

Supplement: FIGURE S2 — Sagittal brain section stained against GFAP (red), vGlut2a-driven GFP (green), and parvalbumin (yellow). (A1,A2) The distribution of vGlut2a-driven GFP shows the extent of the pallial amygdala DM in relation to the dorsal pallium (DP) and integrative olfactory pallium (IOP) both of which lack vGlut2a-driven GFP at large. The tela choroidea is closely attached (white arrows) to the DM an extents up to the olfactory bulb. Also, note that dense population of GFAP-positive fibers surround the anterior commissure similar to the situation in mammals. [file Image_2.JPEG]
