# Supplementary material for: The Zebrafish Amygdaloid Complex – Functional Ground Plan, Molecular Delineation, and Everted Topology
Source: Front Neurosci. 2020 Jul 16;14:608. doi: 10.3389/fnins.2020.00608 (PMC7378821; doi:10.3389/fnins.2020.00608)
Supplement: TABLE S1 — Nomenclature for teleostean telencephalic territories and examples for former labeling inconsistencies. ∗None of the former studies did establish molecularly defined histogenetic units as this study does making it impossible to perfectly compare conflicting interpretations. ∗∗After Biechl and colleagues (Biechl et al., 2017). [file Table_1.DOC]

| **Supplemental Table 1:** Nomenclature discrepancies for telencephalic territories in teleosts. * | | | |
| --- | --- | --- | --- |
| **Nieuwenhuys (2009)** | **Northcutt (2008)** | **Wullimann et al. (1996)** | **This study** |
| **D (dorsal telencephalon) = pallial/cortical territories** | | | |
| Dc (central zone of D) | Dmc (part of pallial amygdala) | Dc = part of amygdala, cortex-homolog (DP – dorsal pallium), and hippocampus (MP) | DP (dorsal pallium = homolog to isocortex = Dc plus anterior pallium) |
| Dl (lateral zone of D) | subdivided | Dl = medial pallium (MP) = mammalian hippocampus | MP (Dl minus pirCtx) |
| Dld (dorsal domain of Dl) | Dld | Dld not recognized |  |
| Dlv (ventral zone of Dl) | Dlv (MP – mammalian hippocampus) | Dlv not recognized (corresponds to Dp of Wullimann) | nLOT (= Dp of Wullimann, and Dlv of Niewenhuys) |
| Dm (medial zone of D) | Dm+Dc = pallial amygdala PMPa/PMCo not recognized | Dm = pallial amygdala  PMPa/PMCo not recognized | DM (anterior Dm) +  PMPa/PMCo (posterior Dm) = pallial amygdala |
| Dp (posterior zone of D) | Dp (main olfactory pallium proper) | Dp not recognized - misinterpreted as part of Dl | IOP (a posterior part of Dl of Wullimann et al., 1996) |
| Nt (nucleus taeniae) | Dlv (anterior) plus  Nt (posterior) | Dp  Nt | nLOT  nLOT GABAergic layer |
| **V (ventral telecephalon) = subpallial/subcortical territories** | | | |
| Vd (dorsal zone of V) | Vd (striatopallidum) | Vd (striatopallidum) | Vd (minus BSTm, BSTa, BSTp, CeAa, CeAl) |
| Vdd (dorsal zone of Vd) | Vdd (striatopallidum) | Vdd not recognized. | CeAa + MeAa |
| Vc (central zone of V) | Vc (not specified) | Vc (not specified) | CeAl + BSTa |
| Vs/Vp (supracommissural/  postcommissural V) | Vs/Vp (central amygdala) | Vs/Vp central/medial amygdala yet unspecified | MeAc + MeAd + MeAv + part of BSTm |
|  |  | Vi (intermediate nucleus of V = mammalian MeA)** | MeAp |
| Vv (ventral zone of V) | Vv (septum) | Vv (septum) | Vv (septum) |
| * Former studies lack molecular definitions for telencephalic territories making precise comparisons challenging.  ** After Biechl et al. (2017). | | | |
